# Self-Accelerating Drops on Silicone-Based Super Liquid-Repellent Surfaces

**DOI:** 10.1021/acsnano.5c04250

**Published:** 2025-06-17

**Authors:** Parham Koochak, Marcus Lin, Ali Afzalifar, Arsalan Hashemi, Sankara Arunachalam, Ayan Shoaib, Valtteri Turkki, Tapio Ala-Nissila, Dan Daniel, Maja Vuckovac, William S. Y. Wong

**Affiliations:** † Department of Applied Physics, School of Science, 174277 Aalto University, FI-02150 Espoo, Finland; ‡ Division of Physical Sciences and Engineering, 216628 King Abdullah University of Science and Technology (KAUST), Thuwal 23955-6900, Saudi Arabia; § MSP Group, Quantum Technology Finland Center of Excellence, Department of Applied Physics, 174277 Aalto University, FI-00076 Espoo, Finland; ∥ Interdisciplinary Centre for Mathematical Modelling and Department of Mathematical Sciences, Loughborough University, Loughborough, Leicestershire LE11 3TU, U.K.

**Keywords:** drop rolling, drop friction, drop adhesion, drop electrification, charge suppression

## Abstract

Design of super liquid-repellent surfaces has relied on an interplay between surface topography and surface energy. Perfluoroalkylated materials are often used, but they are environmentally unsustainable and notorious for building up static charge. Therefore, there is a need for understanding the performance of sustainable low surface energy materials with antistatic properties. Here, we explore drop interactions with perfluoroalkyl- and silicone-based surfaces, focusing on three modes of drop-to-surface interactions. The behavior of drops rolling under gravity is compared to those subjected to lateral and normal forces under constant slide (i.e., friction) and detachment (i.e., adhesion) velocities. We demonstrate that a drop’s characteristic and dynamic mobility depends on surface chemistry, with sequential drop interactions being particularly affected. By utilizing force-and-charge instruments, we show how rolling drops are primarily governed by adhesion and its associated electrostatic effects, instead of friction. Perfluoroalkylated surfaces continuously accumulate charges, while silicone surfaces rapidly saturate. Consequently, sequentially contacting drops accumulate significant charges on the former while rapidly diminishing on the latter. The drop charge suppressing behavior of silicones enhances drop mobility despite their higher surface energy compared to perfluoroalkyls. Quantum mechanical density functional theory calculations show significant differences in surface charge distributions at the atomic level. Simulations suggest that variations in the lifetimes of surface hydroxyl ions likely drive the markedly different drop charging behaviors. Our findings demonstrate the critical role of surface chemistry and its coupled electrostatics in drop mobility, providing valuable insights for designing environmentally friendly, antistatic, super liquid-repellent surfaces.

## Graphical abstract

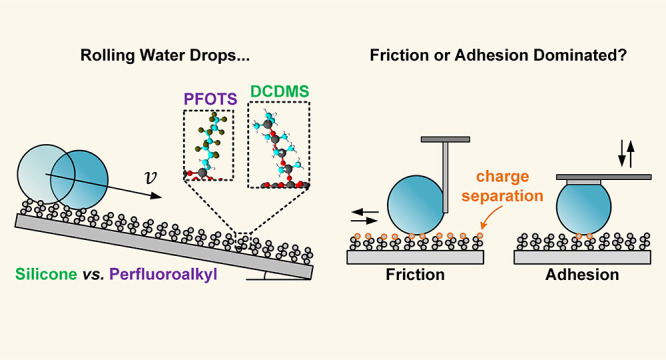

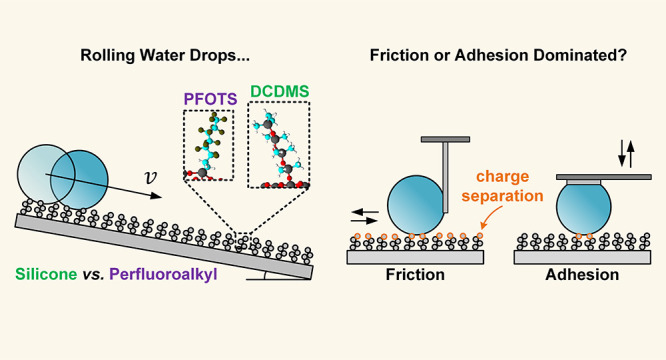

## Introduction

Super liquid-repellent surfaces have enabled many applications requiring high drop or liquid mobility. This includes their use in self-cleaning,^1–3^ antidrag,^4–7^ microfluidics,^8,9^ liquid patterning,^10–12^ and even bubble transport.^13,14^ The development of such surfaces has primarily relied on the combination of surface topographical^15–17^ and surface energy^18–20^ properties. To date, most approaches employ some form of perfluoroalkyl-based chemistry,^21–26^ raising severe environmental and health concerns.^27–31^ Developing fluoro-free surfaces with effective liquid repellency is a key step toward achieving long-term sustainability.^20,32–34^

In contrast to perfluoroalkyl-based surfaces, silicone-based surfaces remain inadequately characterized. Two key distinctions between these variants are currently known. First, the slightly lower surface energy of perfluoroalkylated −CF_2_/–CF_3_ groups compared to dimethyl-silicone (−CH_3_)_2_ groups is well-documented for their lower retention forces.^35–39^ Second, the −CF_2/3_ based surfaces experience significant electrostatic charging during water contact.^40–43^

State-of-the-art slide electrification is typically investigated on hydrophobic surfaces,^33,41,42,44–47^ where drops remain in full solid contact^44,47^ during sliding. For these surfaces, friction is representative of drop sliding mechanics as lateral forces dominate.^48–50^ For super liquid-repellent surfaces (Cassie-state^51^), drops do not experience full solid contact.^52,53^ In these cases, drops roll and capillary bridges form and break (depinning) at the receding side of the drop.^54–56^ Depinning occurs at an angle to the surface, which suggests greater contributions from normal forces.^54^ Electrostatics are also known to influence mobility^41,43,52,53,57^ but remains rarely investigated for the Cassie-state. Consequently, the impact of charge accumulation and friction/adhesion contributions on rolling drops in the Cassie-state is still not entirely clear. This is further complicated by the unknown impact of surface chemistry variations (i.e. dimethyl-silicone) on dynamic wetting (i.e., adaptation, reaction, charging). Here, we comprehensively assess drop mobility, which we collectively classify as rolling, friction, and adhesion interactions. To this end, we address our primary research question: How do hydrophobic surface chemistries and their associated electrostatics influence the rolling behavior of drops on super liquid-repellent surfaces?

In this study, we first analyze the mobility of a naturally rolling drop using both conventional roll-off angle measurements and by analyzing sequentially rolling drops.^54,58^ The former provides a characteristic retention force, while the latter tracks drop mobility and quantifies sequential retention forces across multiple drops. Unlike the static velocity profiles observed on perfluoroalkyl-based surfaces, we find that silicone-based surfaces self-accelerate sequentially rolling drops, achieving up to a 10% increase in speed.

To gain a deeper insight into sequential drop acceleration (or lack thereof), we used force instruments to decouple forces in the lateral^50,59,60^ (i.e., friction) and normal^61^ (i.e., adhesion) axes.^62^ Drops on perfluoroalkyl-based surfaces experience a large increase in friction (ca. 30 times) but minimal variations in adhesion (ca. 97%). Contrasting this, drops on silicone-based surfaces experience a much smaller increase in friction (ca. 3 times) but a significant decrease in adhesion (ca. 50%). By measuring the charges of drops during rolling, friction, and adhesion measurements, we discover that the charge of drops on silicone-based surfaces decreases faster and plateaus at lower values compared to perfluoroalkyl-based surfaces. Trend analysis reveals a strong correlation between rolling, adhesion, and charging dynamics, which together govern the phenomenon of self-accelerating drops rolling on silicone-based surfaces.

In contrast to perfluoroalkyl-based surfaces, silicone-based surfaces appear to experience rapid charge saturation during continuous water contact. To provide a deeper understanding of these molecularly grafted silicone surfaces, we perform quantum mechanical density functional theory (DFT) calculations in combination with molecular dynamics (MD)^63^ simulations. They assess the electronic structure of surfaces and the lifetimes of surface hydroxyl/hydronium ions, respectively. Experimentally observed drop charge suppression behaviors of silicone-based surfaces is corroborated by simulations showing how surface hydroxyl ions are rapidly repelled into bulk water during wetting contact. This leads to increased neutralization despite surface-induced charge separation, effectively reducing Coulombic forces and enhancing drop mobility.

## Results and Discussion

### The Nature of a Rolling Drop: From Friction to Adhesion

Drops can either slide or roll on super liquid-repellent surfaces, depending on their viscosity^64^ and volume.^65^ With water drops, they experience a rolling motion.^66–68^ The contact line of a rolling drop interacts with surface features (Figure 1a, inset) in a manner where both friction-based viscous dissipation^49,69–71^ and adhesion-based depinning^72–74^ dissipation can occur. Together, they govern how drops roll on these surfaces. Here, the study of rolling drops is complemented by coupling friction and adhesion measurements using two custom-built force instruments.

**Figure 1:**
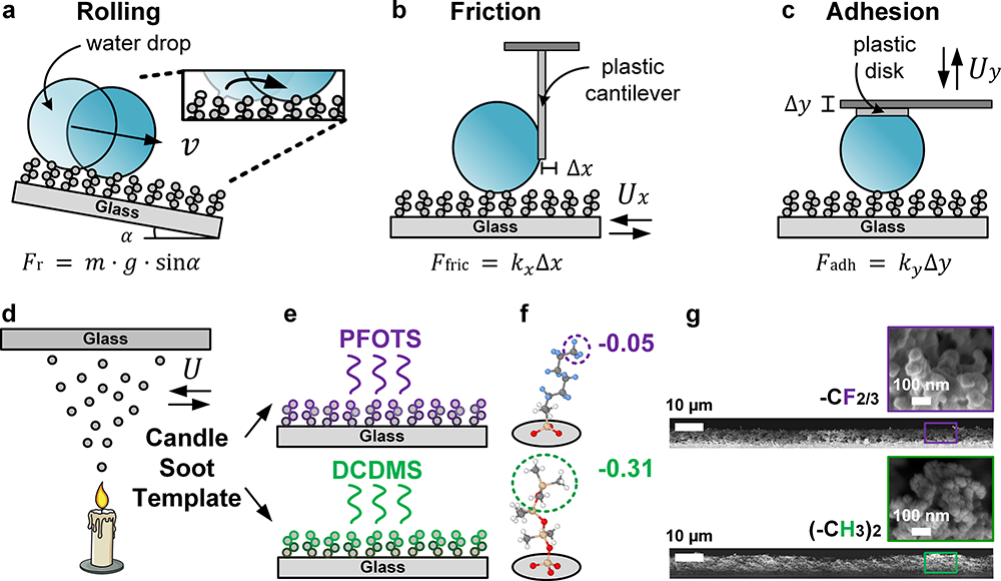
Drop mobility on super liquid-repellent surfaces. (a) Drops rolling on a super liquid-repellent surface, governed by gravity (*g*) and the tilt angle (α). Understanding of rolling is complemented by (b) the lateral motion (Δ*x*) of drops, i.e. friction and (c) the normal motion (Δ*y*) of drops, i.e. adhesion. (d, e, g) Nanoscopically rough but microscopically flat candle soot templated^26^ surfaces are used as model stochastic surfaces. (e–g) Surface chemistry variations include perfluoroalkyl-based (trichloro­(1*H*,1*H*,2*H*,2*H*-perfluorooctyl)­silane: PFOTS, purple) and silicone-based (dichlorodimethylsilane: DCDMS, green) surface functionalization. (f) Schematic representation of the molecular structures, along with charge distribution (in e/chain unit) on their terminal groups, as analyzed using DFT calculations. Charge distribution on the terminal group is analyzed using DFT calculations.

For friction measurements, drops were fixed to a plastic cantilever of known flexural spring constant *k*_*x*_, and dragged along the surface (motion along *x*-axis, while immobile along *y*-axis) at a fixed velocity, *U*_*x*_. The friction force^50,59,60,67^ can be obtained by measuring cantilever deflection Δ*x* (Figure 1b). For adhesion measurements, drops were fixed to a metal cantilever with a known flexural spring constant *k*_*y*_, and brought into contact with the surface (motion along *y*-axis, while immobile along *x*-axis) and detached at a fixed velocity, *U*_*y*_. The pressing force, set at 100 μN, emulates the drop’s weight. The adhesion force^50,59,60,67,75^ is determined by measuring cantilever deflection Δ*y* during detachment (Figure 1c).

To understand the behavior of rolling drops by surface chemistry, we explored two variants of molecularly grafted chemistries: (i) a perfluoroalkyl-based functionalization (Figure 1d,e, purple), via trichloro­(1*H*,1*H*,2*H*,2*H*-perfluorooctyl)­silane (PFOTS), and (ii) a silicone-based^76^ functionalization (Figure 1d,e, green), via dichlorodimethylsilane (DCDMS). Unlike cross-linked bulk silicones,^43,77–79^ i.e. Sylgard 184, DCDMS-based silicones^76^ are known to form chain-like or looplike molecularly thick surfaces composed of dimethyl-silicone. In the following DFT analysis, a terminated dimethyl-silicone chain is used to provide a direct structural comparison to the perfluoroalkyl chain. To provide an atomistic insight into the contrasting chemistries, we analyzed surface interactions with water and mapped surface charge distributions (see Supporting Information, Computational Details). The reported values (Figure 1f) represent an average of over ten different snapshots taken from the last 5 ps of DFT-MD trajectories, where only water molecules are in contact with the surfaces.

Polar water molecules are highly sensitive to the electron density distribution on a surface, which influences both their orientation and proximity to the surface. Regions of high electron density promote hydrogen bond formation, thereby increasing surface hydrophilicity.^80^ Conversely, surfaces with low electron density tend to be more hydrophobic. Surface modification techniques, such as introducing functional groups or applying coatings, can significantly alter the electron distribution, thereby modulating the wettability characteristics of the surface.^81^

Perfluoroalkyl chains induce a slightly higher electron density at the surface compared to alkyl chains (−0.05 *e*/chain, Figures 1f and S1). Surprisingly, DFT calculations predict that terminated dimethyl-silicone chains exhibit a significantly more negative charge, even surpassing that of perfluoroalkyls (−0.31 *e*/chain, Figures 1f and S1). Consistent with experimental literature,^20,32,78^ the water radial distribution functions relative to the surface (Figure S2) indicate that perfluoroalkyl-based surfaces are slightly more hydrophobic than silicone-based surfaces.

Experimentally, both chemistries were developed on a soot-templated nanostructure.^26^ The conductive soot is pyrolyzed, leaving nonconductive silica. The soot-templated structure is unique for its nanoscopically rough but microscopically flat template. This allows for more accurate evaluation using force instruments as microstructural features do not interfere with drop motion.^82,83^ Supplementary SEM and AFM images are included in Figure S3 for reference. Surface functionalization using chemical vapor deposition is expected to achieve near-complete coverage in both instances since reactions are performed to equilibrium, as previously explored.^20^ Perfluoroalkyl- and silicone-based surfaces have thicknesses of ca. 7.4 ± 2 and 12 ± 1 μm, respectively. They are composed of particle grains with sizes of ca. 78 ± 14 and 88 ± 18 nm, respectively (Figure 1g). The characteristic (*F*_r_) and drop-dependent (*F*_r, *i*_) retention forces attributed to each mode of drop interaction (rolling-friction-adhesion) with these surfaces were studied.

### Roll-Off Angle Analysis: Baseline vs Out of Frame Motion

Conventional roll-off angle (α) measurements were first performed to probe characteristic retention forces of surfaces (Figure 2a). This provides a basis of understanding from which dynamic studies are conducted. Drops of 10 μL were placed on grounded surfaces using a grounded metal needle (30G). This eliminates the influence of charging, which we will consider later. The tilting speed varied from 1 to 60 °/min while drops roll off surfaces. α is assessed in two ways. The baseline of the drop is tracked until discernible motion is observed (ca. 10 pixels), capturing the baseline roll-off angle, α_base_ (Figure 2b,c, top row, left axis). Tilting and tracking of the rolling drop continues until the drop rolls beyond the observable field-of-view, with a displacement of *L* ≈ 8 mm. This captures the out-of-frame roll-off angle, α_out_ (Figure 2b,c, bottom row, left axis). By tracking the time between drop-to-surface contact to the respective α_base/out_ event, we obtain the residence time (Figure 2b,c, blue data, right axis) of the sessile drop (*t*_r_) and the time needed for the drop to roll out of the frame.

**Figure 2:**
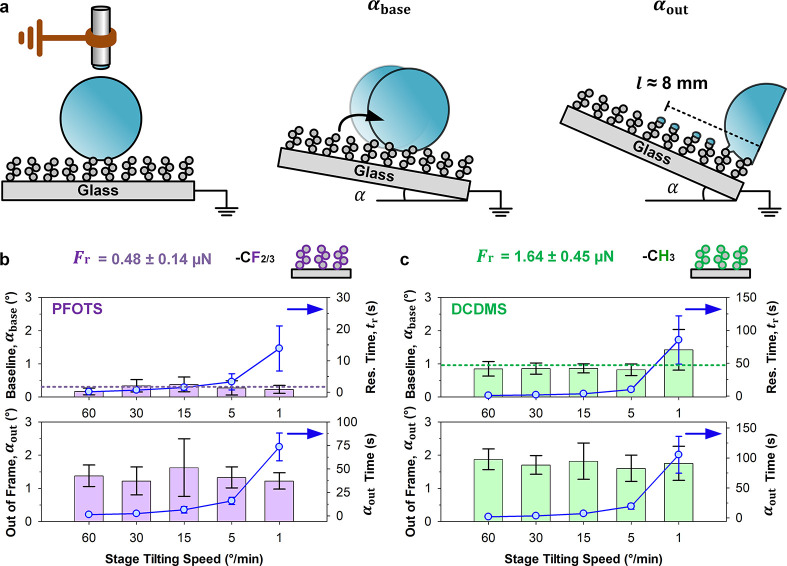
Roll-off angle analysis. (a) Sessile water drops of 10 μL are deposited onto surfaces, before tilting the stage at predefined speeds (1–60°/min) while tracking baseline motion (α_base_) and out-of-frame (α_out_) events. (b, c) Differences between perfluoroalkyl- (purple) and silicone- (green) surfaces are better captured via the baseline method but both illustrate the same trend. Silicone-based functionalization experiences higher α_base_ at high drop residence times, *t*_r_, which may be indicative of adaptation^79,84^ behaviors. *F*_r_ is computed using α_base_ (eq 1). *n* = 5–6, mean ± standard deviation.

Evaluation of α across different tilting speeds reveals a higher retention force (*F*_r_, Figure 1a) for silicone-based surfaces. For α_base_, perfluoroalkyl-based surfaces reach ca. 0.3 ± 0.1° (Figure 2b, top row) while it goes up to ca. 1.0 ± 0.3° (Figure 2c, top row) for silicone-based surfaces. For α_out_, perfluoroalkyl-based surfaces reach ca. 1.4 ± 0.2° (Figure 2b, bottom row) while silicone-based surfaces reach ca. 1.8 ± 0.1° (Figure 2c, bottom row). Both analysis methods show the same trend. α_base_ measurements detect the onset of motion, and are thus averaged (*n* = 25–30, mean ± standard error) for characterizing retention force,

$$F_{r}=mg\sin\;\alpha_{\mathrm{base}}$$ 1

where *m* is the mass of the drop and *g* is the gravitational constant (9.81 m/s^2^). For perfluoroalkyl-based surfaces, *F*_r_ = 0.5 ± 0.1 μN. For silicone-based surfaces, *F*_r_ = 1.6 ± 0.5 μN. Excessive residence time (*t*_r_ > 50 s) by drops on silicone-based surfaces appears to increase α_base_ (Figure 2c, top row) but does not significantly impact α_out_. Speculatively, this may be attributed to the adaptation of silicone-based chains (i.e., water absorption)^84^ under water contact, thereby increasing retention forces.^34,78,79^ In the following dynamic analysis, we kept location-specific contact time significantly below this time scale (≪50 s) to avoid this effect. To compare results from different experiments (e.g., rolling-to-friction-to-adhesion), we also report both absolute (*F*_r,*i*_) and its corresponding dimensionless retention force,

$$F_{r,i}\left(-\right)=F_{r,i}/\gamma D$$ 2

where γ = 0.072 N/m, and *D* is the diameter (±0.01 mm) based on drop volume (6 or 10 μL).

### Dynamic Roll-Off Analysis: Racing Drops

To provide additional insights beyond the statically measured characteristic retention force (*F*_r,*i*_), sequentially rolling drops are analyzed for dynamic retention forces (Figure 3a). 40 drops were dispensed at a drop interval (d*t*) of 10 s (Movie S1). They roll on surfaces under a fixed pretilt angle (α = 2°). Drop velocities (*v*) are effectively controlled by surfaces (Figure 3b,c) under gravity-driven acceleration (*a*). The maximum drop velocity (*v*_m_) of each drop (nonterminal) was extracted over predefined contact lengths, from *L* = 5 mm to *L* = 25 mm (Figure 3a,d). At short contact lengths, drops on both perfluoroalkyl- and silicone- surfaces reach similar maximum velocities (Figure 3d, *L* = 5–10 mm).

**Figure 3:**
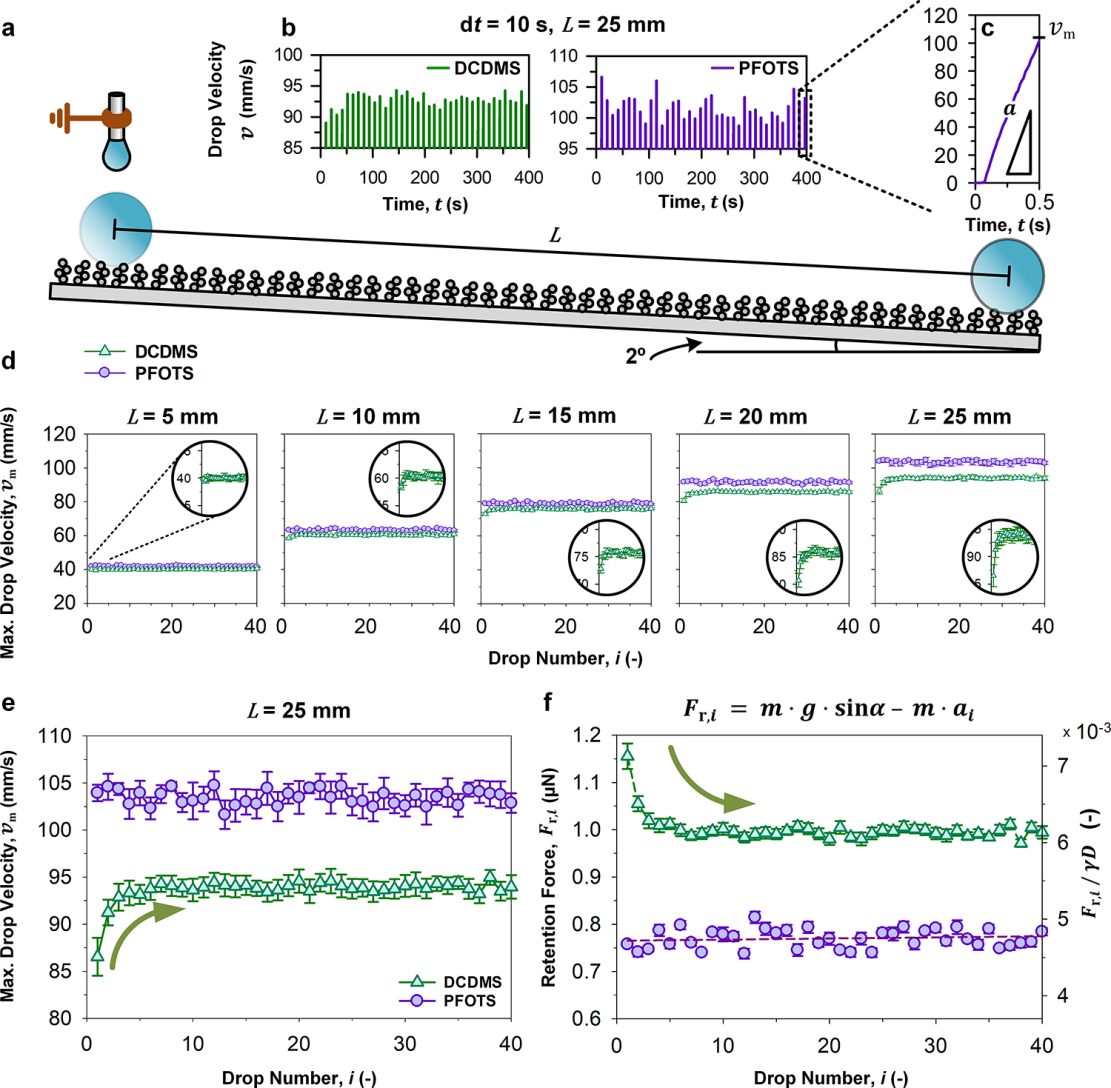
Rolling drop analysis. (a) Using pretilted and ungrounded surfaces (α = 2°), sequential water drops (6 μL) are dispensed off a grounded 30G needle. The needle-and-drop are not in contact with the surface. Drops land without significant bouncing or liftoff. Therefore, (b, c) drops accelerate linearly until they roll off surfaces: (c) As illustrated by a perfluoroalkyl-based surface. (d) The maximum drop velocity is tracked over different contact lengths, from (d) *L* = 5 mm to *L* = 25 mm. (e) At *L* = 25 mm, drop roll-off behaviors are magnified for improved visualization. (f) Dynamic retention force per drop is computed alongside the dimensionless retention force (eq 2). *n* = 6, with mean ± standard error.

As expected, when drops roll further (Figure 3d, *L* = 15–25 mm), they reach higher maximum velocities (*v*_m_) on perfluoroalkyl- surfaces. Nonetheless, no evident velocity trends are observed alongside sequential drops, with *v*_m_ of 104 ± 1.0 mm/s at *i* = 1, to 103 ± 1.0 mm/s at equilibrium (*i* = ∞). Despite the lack of an overall trend, interdrop velocities are more variable, with Δ*v* ranging from 0.06 mm/s up to 3.1 mm/s (Figure 3e, *L* = 25 mm, purple). This suggests that retention forces do sporadically vary, but neither increase nor decrease. With a travel time of ca. 0.48 s over 25 mm, the average acceleration is stable at ca. 216 mm/s^2^ between drops (Figure S4).

On the contrary, drops on silicone- surfaces experience increasing maximum velocities over the first 10 drops before reaching equilibrium (Figure 3e, *L* = 25 mm, green). On average, the maximum velocity increases from 87 ± 2.0 mm/s at *i* = 1, up to 94 ± 0.4 mm/s at *i* = ∞. As drops do not decelerate, we can safely rule out the influence of both adaptation^79,84^ and degradation.^85^ In addition, the contact length (*L*) dependent acceleration (Figure 3d, *L* = 5–25 mm, green) is indicative of the subtle effects behind surface charge,^46^ which we will study in the final section (The Charge-Based Origins of Force Variations). With a travel time of ca. 0.58 s (*i* = 1) and 0.54 s (*i* = ∞) over 25 mm, drops accelerated at ca. 150 and 176 mm/s^2^, respectively. These drop-dependent (*i*) differential behaviors in rolling are rationalized using a net force balance, which is a function of the net acceleration of the *i* ^th^ drop (*a*_*i*_),

$$F_{\mathrm{net},i}=ma_{i}=mg\sin\;\alpha -F_{r,i}$$ 3

where *F*_r,*i*_ is the drop-dependent retention force. Rearranging eq 3 gives,

$$F_{r,i}=m(g\sin\;\alpha -a_{i})$$ 4

When *i* = 1, *F*_r,*i*_ is the characteristic retention force (*F*_r_). When *i* > 1, dynamic influences from adaptation,^79,84^ degradation,^85^ charging,^23,24,42,46^ etc. may occur, leading to changes in retention forces. Using eq 4, perfluoroalkyl-based surfaces show a *F*_r,1_ ≈ *F*_r,∞_ ≈ 0.76 μN for a 6 μL drop (Figure 3f, purple data). Contrasting this, silicone-based surfaces show a *F*_r,1_ ≈ 1.15 μN and *F*_r,∞_ ≈ 1.00 μN for a 6 μL drop. The retention force refers to how a surface resists the rolling motion of a drop. It is impacted by surface chemistry, topography, geometrical contact, and any derivative surface charging, adaptation, and/or degradation effects. By studying the retention force variations between drops, we observe their cumulative influence on drop-to-surface interactions. Using a different drop interval (d*t* = 2.5 s), we observe nearly identical trends for both silicone-based and perfluoroalkyl-based surfaces (Figures S5–S7), demonstrating time-independent behavior within our domain of investigation.

### Dragging Drops: Drop Friction Analysis (Forces Lateral to Surface)

To understand how lateral forces (along the *x*-axis) influence drop rolling mobility, we measure drop friction on ungrounded surfaces. Water drops of 10 μL were immobilized using a plastic cantilever and slid across surfaces at a fixed velocity, *U*_*x*_.^83^ The drop is pinned to the surface-etched cantilever to prevent contact line motion during measurements. The drop is dragged across the surface for a contact length of *L* = 10 mm at a slow drop sliding^45,83,86^ velocity, *U*_*x*_ = 1 mm/s to avoid transitional behaviors^83,86^ in drop friction. During sliding (push or pull), the cantilever that holds the drop deflects, allowing for in situ force measurements. A larger deflection indicates a higher lateral force exerted on the drop by the surface. After sliding across *L* = 10 mm, the drop reverses on the same path back to its point of origin (Figure 4a). Drops and surfaces are electrically insulated (ungrounded) in these experiments. A drop sliding interval (d*t*) of 10 s, is defined as the time taken for a drop to revisit the center of the track (Movie S2).

**Figure 4:**
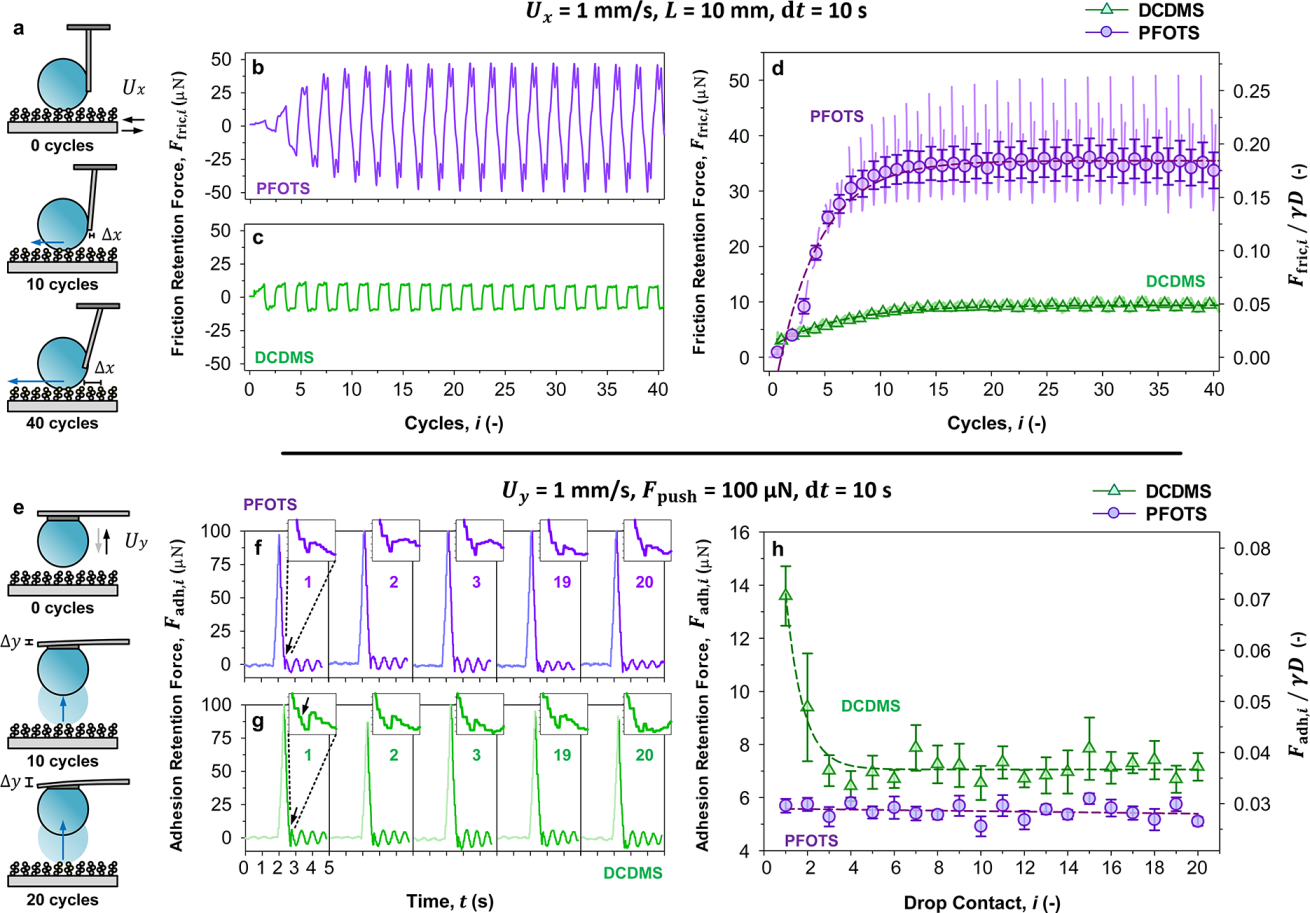
Drop friction vs drop adhesion analysis. *Friction:* (a) Drops are dragged on surfaces by a plastic cantilever, with changes in lateral forces measured by cantilever deflection (Δ*x*). Friction force measurements are performed for (b) perfluoroalkyl- and (c) silicone- based surfaces and collated as force vs cycles. (d) The build-up of friction force eventually results in a crossover of *F*_fric_ between silicone- and perfluoroalkyl- based surfaces. *Adhesion:* (e) Drops are contacted with surfaces using a metal cantilever and a plastic drop holder. Adhesion force measurements are performed for (f) perfluoroalkyl- and (g) silicone- based surfaces and collated as force vs unit time. During sequential drop contact-and-departure (i.e., *i* = 1, 2, 3, 19, and 20), the cantilever holding the drop is pulled downward before detachment, with deflection (Δ*y*) measuring the (f, g) so-termed pull-off force or adhesion force (from black arrow to equilibrium deflection). Insets have a time domain of 0.5 s and a force domain of 30 μN. (h) Pull-off force curves show the steady adhesion force of perfluoroalkyl-based surfaces and the decreasing adhesion force of silicone-based surfaces over sequential contact. Dimensionless forces are also presented as *F*_fric_/γ*D* or *F*_adh_/γ*D*. *n* = 3 and 5, respectively, with mean ± standard error.

The friction-based retention force is determined from the plateaus in the friction retention force curves (Figure 4b,c). The characteristic friction-based retention forces, *F*_fric,1_, were measured as 0.99 ± 0.50 μN for perfluoroalkyl-based surfaces and 3.08 ± 0.18 μN for silicone-based surfaces. At equilibrium, the friction-based retention forces, *F*_fric,∞_, reached 35 ± 3.4 and 8.9 ± 0.16 μN, respectively. Notably, drops on perfluoroalkyl-based surfaces exhibit a rapid increase in friction force with sequential sliding cycles. By the second cycle, the friction force matches that of silicone-based surfaces and surpasses it entirely by the third cycle (Figure 4d). Notably, while perfluoroalkyl-based surfaces have slightly lower initial friction force (*F*_fric,1_) compared to silicone-based surfaces, the latter outperforms the former by almost 3 times lower equilibrium friction force (*F*_fric,∞_). Increase in friction may be attributed to adaptation^79,84^ or degradation^85,87^ events. However, performing an electrical discharge (Figure S8) via a wire-to-drop connection midway through 10 cycles restores surfaces retention forces back to their original levels (i.e., at first cycle). This suggests that friction force variations are likely attributed to surface charge, which we will examine in the final section (The Charge-Based Origins of Force Variations).

### Detaching Drops: Drop Adhesion Analysis (Normal to Surface)

To understand how normal forces (along the *y*-axis) influence drop rolling mobility, we measured drop adhesion on surfaces. A 10 μL drop is placed on a plastic holder, attached to a metal cantilever, and brought into contact with surfaces and detached (Figure 4e) at a constant velocity, *U*_*y*_.^61^ The drop is pinned to the edges of the plastic disk to prevent contact line motion during measurements. The drop weight is emulated by predefined compression (*F*_push_ = 100 μN). Drops and surfaces are electrically insulated (ungrounded) in these experiments. A slow approach-contact-detachment velocity of 1 mm/s prevents vibration-induced noise and velocity-induced transitions during measurements (Figure S9). During adhesion measurements, the cantilever deflects (Δ*y*) as drops detach from surfaces. A larger deflection indicates a higher normal force exerted on the drop by the surface. The pull-off event occurs right before drop detachment (Figure 4f–g, inset, arrow). The drop contact interval (d*t*) of 10 s, is defined as the time taken between the point of pull-off and recontact (Movie S3). The adhesion-based retention forces are measured as the pull-off force from adhesion force curves^61^ (Figure 4h). The characteristic adhesion-based retention forces, *F*_adh,1_, were measured as 5.7 ± 0.3 μN for perfluoroalkyl-based surfaces and 13.6 ± 1.1 μN for silicone-based surfaces. At equilibrium, the adhesion-based retention forces, *F*_adh,∞_, were measured at 5.4 ± 0.3 and 7.1 ± 0.4 μN, respectively.

Perfluoroalkyl-based surfaces do not show variation across sequential contact-detachment cycles (Figure 4h, purple). Contrasting this, adhesion forces on silicone-based surfaces decrease significantly during the first 3 contacts (Figure 4h, green). Notably, while perfluoroalkyl-based surfaces have much lower initial adhesion (*F*_adh,1_) compared to silicone-based surfaces, drop adhesion on the latter closely approaches the former by the third sequential contact (*F*_fric,3_). Since adhesion forces did not increase over sequential contact cycles, we can confidently rule out the influence of adaptation,^79,84^ and degradation.^85,87^ By process of elimination, we attribute this decrease in adhesion force to surface charge suppression, which we will examine in the final section (The Charge-Based Origins of Force Variations).

### Lateral vs Normal Forces and Its Collective Impact on Net Drop Mobility

Collectively, drop mobility of water on silicone-based and perfluoroalkyl-based surfaces clearly differs across methods employed. Forces measured on surface variants are represented in both absolute and dimensionless forms. A summary of the absolute and dimensionless forms under initial (*i* = 1) and equilibrium behaviors (*i* = ∞, first order fitting) is included in Table S1. To understand characteristic trends, we introduce the force correlation coefficient, *F*_∞_/*F*_1_, which represents the net change in forces acting on drops using the 3 methods used. By analyzing rolling, friction, and adhesion behaviors, we decouple lateral and normal retention forces that control the motion of a rolling drop, providing greater insight into how they roll. Curiously, the coefficient aligns well between rolling and adhesion measurements, while acting opposite for rolling and friction measurements (Table 1).

**Table 1:** Force Correlation Coefficient: Initial (*i*0) vs Equilibrium (∞)

	PFOTS	DCDMS
force method (*F*_r*,i*_)	*F*_∞_/*F*_*i*0_	*F*_∞_/*F*_*i*0_
rolling	1.01	0.85
friction	36.1	3.04
adhesion	0.97	0.52

In addition to visual similarities, it is sometimes suggested^67,82,88,89^ that friction measurements are representative of rolling drops. In this work, we found a surprisingly good match between the coefficients for rolling and adhesion, while a significant discrepancy exists between rolling and friction (Table 1). We attribute this to depinning dissipation arising from capillary bridge breakage^54–56,72^ at the receding side of a rolling drop (Figure 1a, inset). Depinning forces dominate locally with a large normal force vector, leading to significant contributions to the retention force during rolling. While this qualitatively indicates the dominant influence of adhesion and normal force contributions on a rolling drop, improved quantification of these effects may be worth exploring in future studies (see Conclusions).

### The Charge-Based Origins of Force Variations

To better understand the behavior of adhesion-dominated rolling of drops on perfluoroalkyl- and silicone-based surfaces, we complemented drop rolling-friction-adhesion measurements using drop charge measurements. With rolling drops, a platinum wire catches and discharges^41,42,73^ them into an electrometer (Figure 5a).^41,42,46^ With friction measurements, the plastic cantilever is replaced with a metal cantilever, which is connected to an electrometer (Figure 5b). With adhesion measurements, a platinum wire is placed directly inside the drop while live force and charge measurements are performed (Figure 5c). Rolling-adhesion charge measurements are semicontinuous while friction charge measurements are continuous (see Supporting Information, Tables S2 and S3). The latter can result in larger magnitudes (per unit area) compared to those accumulated by rolling-adhesion drops.^45^

**Figure 5:**
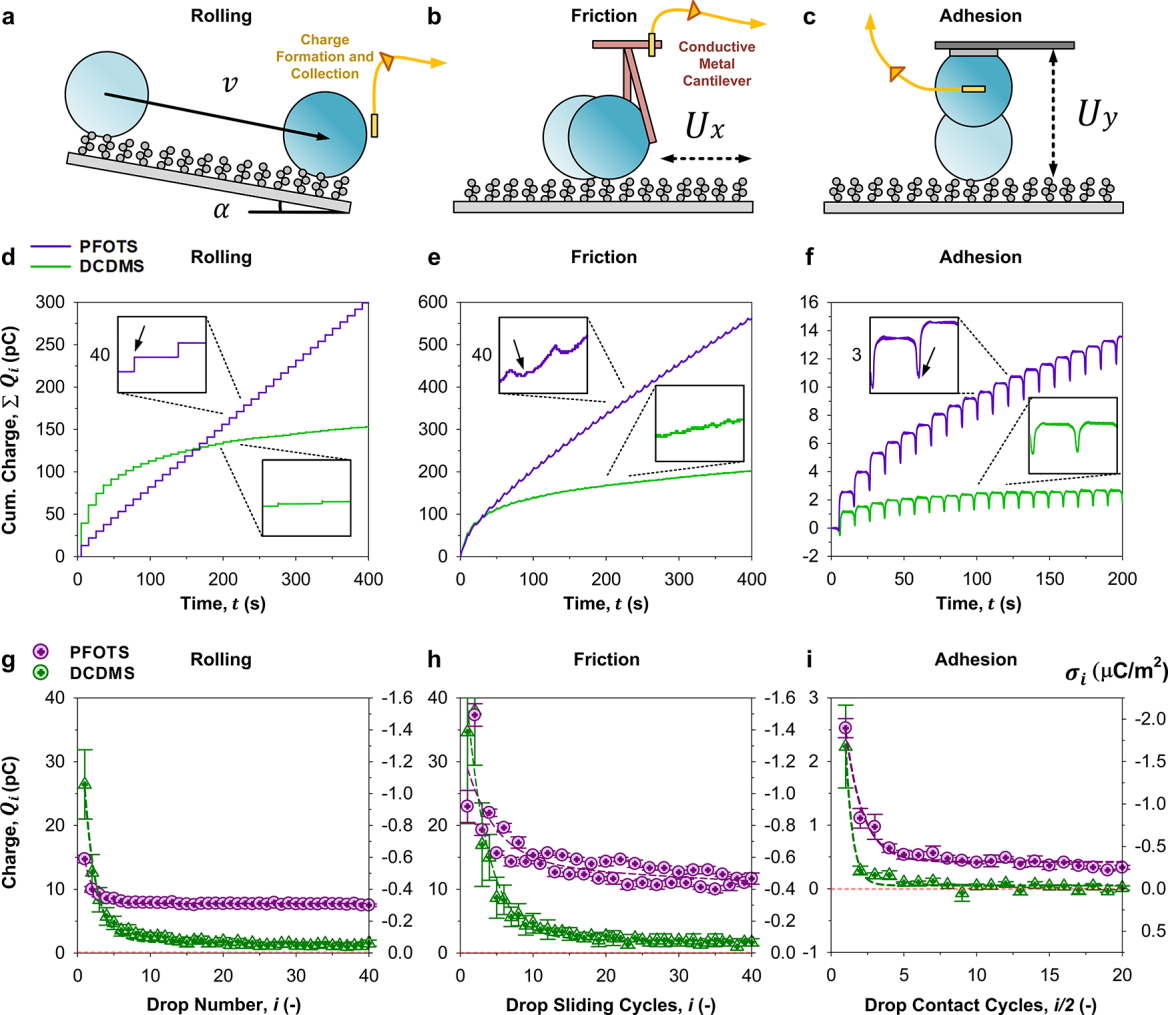
Drop rolling-friction-adhesion: from mobility to charging. Force measurements (See Figure S10) are (d–f) paired to charge measurements via electrometric measurements. A platinum wire (150 μm, 99.99+%) is used to (a, d, g) collect rolling drops’ charges, (b, e, h) measure live slide-friction drops’ charges, and (c, f, i) collect contact-adhesion drops’ charges. (d–f) Cumulative (∑*Q*_*i*_) and (g–i) differential (*Q*_*i*_) measurements are presented alongside geometrical-area based surface charge density, σ_*i*_ (g–i), right axis). Repeats and averaging of cumulative charge curves in (d–f) are also included in Figure S11. *n* = 3–5, with mean ± standard error.

The presence of the platinum wire may influence absolute friction and adhesion force measurements (see Supplementary Discussion and Figure S10). However, it enables in-drop probing of surface charge effects. In addition, as the electrometer is directly connected to the drop, the drop remains neutral throughout the measurements. Contact with surfaces therefore allows the drop to detect charges that are present on the surface. The dynamics of sequential drop charge collection differs within each method.

With rolling drop measurements, sequential drops are initially neutral. As they roll, they collect charges, eventually depositing it as a single pulse into the electrometer via the platinum wire. This produces the semicontinuous and sequential step-function that is observed during cumulative charge collection (Figure 5d, inset, arrow). This represents the positive charges that are collected by each fresh drop, with the corresponding negative charges left behind on surfaces. For perfluoroalkyl-based surfaces, net positive drop charging barely slows and does not saturate. For silicone-based surfaces, net positive drop charging reaches equilibrium by the 20th cycle, where sequential drops remain almost neutral.

In friction measurements, the wire, drop, and surfaces are continuously connected. As drops are dragged across the surfaces, they charge positively, with charges promptly collected by the electrometer. Meanwhile, the surfaces charge negatively. Charge separation is known to occur at the receding contact line.^44^ As drops reverse along their paths, neutral drops detect the negatively charged surfaces. These recently deposited negative charges flow into the drop and to the electrometer. For perfluoroalkyl-based surfaces, this results in brief surface neutralization events (ca. 20%), where charges detected become slightly negative before again increasing (Figure 5e, inset, arrow). The negative surface charge detection event becomes more prominent beyond the fifth cycle, which is indicative of surface charge accumulation. Net positive drop charging slows but does not saturate. For silicone-based surfaces, the negative-to-positive charging dynamics is similar but occurs at much smaller magnitudes. Net positive drop charging reaches equilibrium by the 20th cycle, where sequential drop interaction cycles remain almost neutral.

In adhesion measurements, the wire and drop are intermittently connected to the surfaces. The surfaces make semicontinuous contact during drop interaction and detachment. As the drop detaches, it acquires a positive charge, leaving the surface negatively charged. Upon recontact, negative charges on the surface flow into the drop (Figure 5f, inset, arrow), neutralizing the surface. Adhesion-based charge separation exhibits the most unique surface charging-neutralization curves, as detachment involves pulling a drop off the surface. This process is entirely governed by receding contact lines, after which drops reacquire a positive charge. Over sequential drop contacts, we evaluated the net accumulation of positive charges using an electrometer. For perfluoroalkyl-based surfaces, net positive drop charging slows but does not reach equilibrium. For silicone-based surfaces, net positive drop charging reaches equilibrium by the fifth cycle, where drop interaction cycles no longer acquire a net positive charge.

Differentiation of the cumulative charge measurements (Figure 5g-i) provides an analysis of charge per drop interaction, *Q*_*i*_ . As before, to elucidate characteristic trends, we introduce the charge correlation coefficient, *Q*_∞_/*Q*_max_ (Table 2), which represents the ratio of the equilibrium charge to the maximum charge generated during drop-to-surface interactions.

**Table 2:** Charge Correlation Coefficient: Initial (*i*0/max) vs Equilibrium (∞)

Surfaces	PFOTS			DCDMS		
Charge method (*Q*_i_)	*Q*_max_	*Q*_∞_	*Q*_∞_/*Q*_max_	*Q*_max_	*Q*_∞_	*Q*_∞_/*Q*_max_
Rolling, *Q*_r,i_ (pC)	15 ± 0.6	7.5 ± 0.2	0.5	26 ± 5	1.5 ± 0.5	0.06
Friction, *Q*_fric,i_ (pC)	37 ± 2	12 ± 1	0.33	35 ± 14	1.6 ± 0.6	0.05
Adhesion, *Q*_adh,i_ (pC)	2.5 ± 0.2	0.34 ± 0.03	0.14	2.2 ± 0.7	0.01 ± 0.03	0.005

With rolling drops and friction measurements (Figure 5g,h), charge correlation coefficient on silicone-based surfaces decreases rapidly and to a much larger extent (10^–2^ vs 10^–1^) than perfluoroalkyl-based surfaces. With adhesion measurements, this difference becomes even more drastic, with the charge correlation coefficient reaching 10^–3^ vs 10^–1^ for silicone-based and perfluoroalkyl-based surfaces, respectively. Notably, charge correlation behavior is very similar between rolling and friction (Table 2), demonstrating how the liquid-contact triboelectric phenomenon of rolling drops is still highly correlated to the so-termed slide electrification.^46^ Drop charge suppression in adhesion measurements reveal the largest contrast (Figure 5i and Table 2) between perfluoroalkyl-based and silicone-based surfaces.

When normalized^46,47,90^ by the respective geometrical contact area (Rolling: ca. 25 mm^2^, Friction: ca. 10 mm^2^, and Adhesion: ca. 1.3 mm^2^), the corresponding average surface charge density (σ)^91^ is shown in Figure 5g–i (right axes). In this work, we report the contact domain-averaged charging behaviors. Previous studies have shown how surface potential,^91^ charge accumulation,^46^ and forces^41^ are nonlinearly influenced by long slide lengths. Here, we have employed comparatively short contact lengths, which limit the impact of nonlinear behaviors that are prevalent for longer contact distances.^41,46,91^

Across all drop mobility measurements, the drop charge suppression exhibited by silicone-based surfaces is at least 1 order of magnitude greater than that observed on perfluoroalkyl-based surfaces (Table 2). To understand the influence of charges on retention forces and drop mobility, we re-evaluate rolling drops. As a revision to eqs 3 and 4, we attribute sequential drop acceleration only to electrostatically induced (*e*) forces, *F*_e_(*i*),

$$F_{\mathrm{net},i}=ma_{i}=mg\sin\;\alpha -F_{r,1}-F_{e}(i)$$ 5

With silicone-based surfaces, we have previously observed a decrease of ca. 0.17 μN (Figure 3f) for a 6 μL drop. Since *mg* sin α and *F*_r,1_ are constants, we attribute changes in *F*_net,*i*_ to charge-induced effects. Hence, a decreasing *F*_e_(*i* = ∞) induces the corresponding decrease in retention force, as charge suppression progresses.

It is known that sequentially rolling drops can experience charge-induced forces due to the presence of surface-deposited charges.^41,57^ These attractive forces lead to either acceleration or deceleration, depending on the proximity between the charged drop and the surface.^41^ According to Coulomb’s law, the electrostatic force (*F*_e_) between two points is given by

$$F_{e}\left(i\right)=k_{e}\frac{Q_{d}\left(i\right)Q_{s}\left(i\right)}{d^{2}}$$ 6

where *k*_e_ is Coulomb’s constant, *Q*_d_(*i*) and *Q*_s_(*i*) are the respective drop and surface charges, with *d* the distance between them.

For perfluoroalkyl-based surfaces, the first rolling drops charge up strongly, while subsequent drops charge less, though they do not fall below 50% of the charge of the first drop. This results in sustained accumulation of charges on the surface. Consequently, strong electrostatic forces between the surface and the drop persist, which qualitatively explains the sporadic nature of rolling velocities observed (Figure 3e,f, purple data). For silicone-based surfaces, the first rolling drops charge up strongly, while subsequent drops charge significantly less, falling to just 6% of the charge of the first drop (Figure 5g). This trend is mirrored in both friction and adhesion measurements, with drops charging at 5% and 0.5% of the first drop, respectively (Figure 5h,i). This lowers the extent of charge accumulation on the surface as well. As a result, electrostatic forces between the surface and the *i* ^th^ drop weaken over sequential rolling drops. Since rolling drops on silicone-based surfaces are predominantly controlled by adhesion (Table 1), which is in turn significantly influenced by electrostatics (Table 2), drop charge suppression enhances sequential drop mobility, leading to electrostatically uninhibited acceleration.

### Correlating Drop Mobility-and-Charge Behaviors

To provide a quantification of behaviors beyond the correlation coefficients (Tables 1 and 2), we study force and charge decay behaviors for silicone-based surfaces using normalized force (*F̂*_i_) and charge (*Q̂*_i_) with rolling and adhesion measurements. Friction measurements are not studied here as it contrasts drop rolling mobility behaviors, and likely remains a minor contributor (Table 1). We model the force and charge behaviors using first-order kinetics, following adaptive^79,84^ and reactive wetting^85,87^ models, and generalize the parameter of *ŷ*_i_. Here, we revise the start of *i* from 1 (count) to 0 (native),

$${\hat{y}}_{i}=(1-{\hat{y}}_{\infty })e^{-\lambda (i-1)}+{\hat{y}}_{\infty }$$ 7

where *ŷ*_∞_ is the equilibrium force (*F̂*_∞_) or charge (*Q̂*_∞_), and λ is the time-dependent discharge/force decay parameter, where λ­(*i* – 1) = *f*(*t*, τ). λ is a function of both drop interaction history (*i* or *t*) and the surface time constant (τ). The former is an experimental parameter (i.e., drop number or drop interval) while the latter is a function of surface properties. Further details are provided in the Supplementary Discussion. We use λ, *ŷ*_∞_ and cross model fitting (*R* ^2^ analysis) to assess correlations between the decay of force and charge (Figure 6a,b, triangles and circles). Fitting the model (eq 7) to the respective rolling/adhesion and force/charge measurements, we define the best-fit models (Figure 6a,b, dashed lines) at high *R* ^2^ values (0.91 and 0.93, respectively).

**Figure 6:**
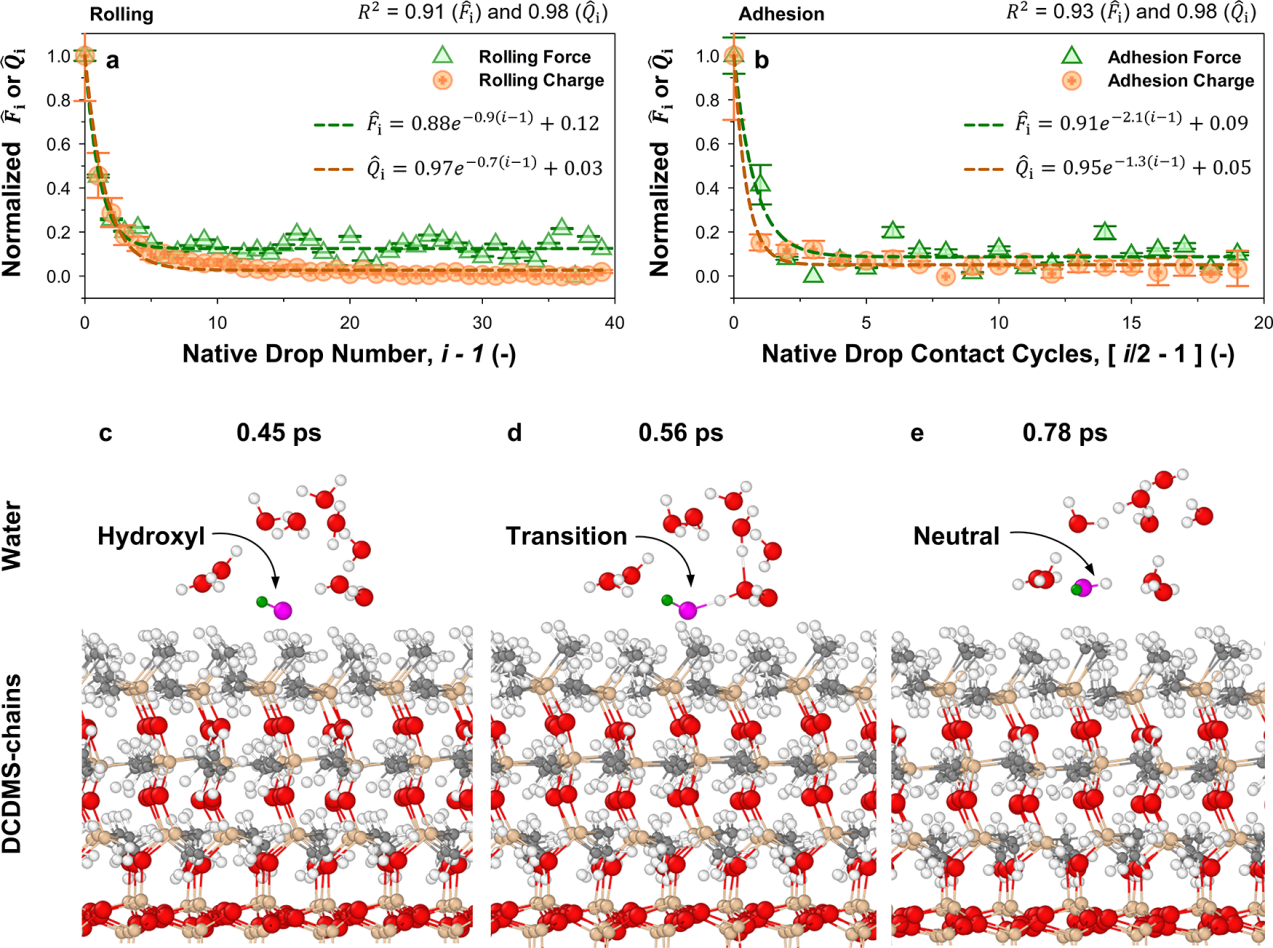
Charge-to-mobility correlations and DFT-MD simulations. Normalized force and charge measurements are analyzed using first-order kinetics (eq 7) to study correlations in parametric decay behavior between (a) rolling drops and (b) drop adhesion on silicone. *n* = 5, mean ± standard error. DFT-MD: Structure of hydroxyl ion and water molecules near the DCDMS-terminated surface. (c) 0.11 ps before water formation, (d) at the moment of O–H bond formation, and (e) 0.22 ps after water formation. Red, white, cyan, gray, and orange spheres represent O, H, F, C, and Si atoms, respectively. The oxygen and hydrogen atoms of the hydroxyl ion are highlighted in pink and dark-green for clarity. For better visualization, extra water molecules were removed from the DFT-MD snapshots.

We note similarities in λ, with rolling at 0.7–0.9 and adhesion at 1.3–2.1. A higher λ represents a faster decay in (*F̂*_∞_) or charge (*Q̂*_∞_), which corresponds well with current observations. Analysis of single- and cross-model fits corroborates how force correlates with charge, moderately (*R* ^2^ > 0.55) in rolling dynamics and strongly (*R* ^2^ > 0.85) in adhesion dynamics, respectively (Table 3).

**Table 3:** Single- (bold) and Cross- (underlined) Model Fitting to First-Order Kinetics

*R*^2^ analysis	Rolling		Adhesion	
Fitted Model	Force	Charge	Force	Charge
Force (*F̂_i_*)	**0.91**	0.56	**0.93**	0.85
Charge (*Q̂_i_*)	0.70	**0.98**	0.91	**0.98**

### Origins of Drop Charge Suppression

To investigate the origins of drop charge suppression, we performed DFT-MD simulations to elucidate the interactions of hydronium (H_3_O^+^) and hydroxyl (OH^–^) ions with the two surface variants. Building on prior studies showing that water readily dissociates into H_3_O^+^ and OH^–^ at interfaces,^92–94^ we modeled drop-to-surface contact by first equilibrating the system with pure water. Subsequently, water molecules closest to the molecularly grafted surfaces were selectively converted to hydronium/hydroxyl and hydroxyl/hydronium ions, ensuring that the overall system remained at a neutral charge.

Our DFT-MD simulations reveal distinct behaviors for hydronium and hydroxyl ions at the respective interfaces. Hydronium ions exhibit similar interactions with both surface variants, showing a strong preference for the transfer of excess protons into the bulk water phase, indicative of interfacial instability (see Movie S4). Contrasting this, hydroxyl ions are more transiently stable. We define the hydroxyl ion lifetime as the duration it remains unreacted prior to protonation and subsequent water formation (see Movie S5).

On silicone-grafted surfaces, the hydroxyl remains parallel with the hydrogen atom slightly oriented outward (Figure 6c). Water formation through protonation begins at 0.56 ps and completing the transition by 0.78 ps (Figure 6d,e). On perfluoroalkyl-grafted surfaces, the hydroxyl remains temporarily adsorbed for up to 2.8 ps before proton affinity drives a transition that results in water formation (Figure S12). Silicone-grafted surfaces show a shorter hydroxyl ion lifetime than for perfluoroalkyl-grafted surfaces. This is attributed to the high local molecular charge density, which enhances Coulombic repulsion, causing the hydroxyl ion to remain at the interface for much shorter time periods. During water formation, the emergence of water clusters (Movies S4 and S5) that mediate proton transfer becomes particularly prominent (Figure 6c). Following protonation, water molecules at the interface quickly stabilize and become well-isolated (Figure 6d,e), while newly formed hydroxyl groups migrate into the bulk through these clusters.

Our findings align with partial charge calculations (Figures 1f and S1), which show that silicone-based surfaces exhibit higher electronegativity compared to perfluoroalkyl-based surfaces, leading to greater repulsion of OH^–^ ions. Furthermore, a comparison of the frontier orbital energies of grafted perfluoroalkyl groups (HOMO = −3.27 eV, LUMO = 2.33 eV) and terminal dimethyl-silicones (HOMO = −2.27 eV, LUMO = 2.95 eV) suggests that electrons in silicone-based surfaces are more energetic in both occupied and unoccupied states. This results in weaker electron-withdrawing properties, further destabilizing OH^–^ ions at the interface. Overall, while hydronium ions remain unstable on both surfaces, the faster migration of hydroxyl ions into the bulk neutralizes drop charges at a higher rate for silicone-based surfaces. This behavior provides critical insight into the as-observed charge suppression phenomena.

In summary, our combined experimental observations of drop mobility and charge, supported by DFT-MD simulations, provide strong evidence for the underlying molecular mechanisms driving drop charge suppression. The dynamic suppression of drop charges during sequential drop interactions eliminates drop-to-surface Coulombic interactions, enabling electrostatically uninhibited drop acceleration. In this work, we have demonstrated the use of DFT-MD simulations to predict relative charge suppression behaviors of two iconic hydrophobic chemistries. In the future, we foresee the use of high-throughput DFT-MD calculations for investigating complex adaptive chemistries^42^ or mixed molecular surfaces^43^ for achieving molecularly engineered charge control.

## Conclusions

Designing fluoro-free super liquid-repellent surfaces that suppress drop rolling electrification is beneficial for use in electronics manufacturing and cleanroom environments. Using model nanostructured surface topographies, we have demonstrated that this can be achieved through a one-step silicone-based surface chemistry modification. Using drop mobility (rolling-friction-adhesion) analysis and drop charge measurements, we discovered that silicone-based surfaces exhibit enhanced drop mobility and drop charge suppression during sequential drop interactions. DFT-MD simulations describe how dimethyl-silicone chains readily repel surface-bound hydroxyl groups back into the bulk of the contacting water, thereby enabling drop neutralization. As a result, a significant suppression of drop charge (up to 3 orders of magnitude) correlated with the decreasing adhesion forces observed. Consequentially, rolling drops, which are predominantly influenced by adhesion, accelerate and enhance their own mobility simply by rolling along the same track. Contrasting this, perfluoroalkyl-based surfaces experience charge accumulation on both drops an surfaces, which leads to sporadic and even negatively impact drop mobility (i.e., friction measurements). This work provides new insights into molecularly grafted nanostructured surfaces that suppress drop electrification while enhancing adhesion-dominated rolling dynamics. We also invoke new research questions: (1) How would model^44,45^ measurements change if conducted at the natural rolling velocity (80–100 mm/s)? (2) How, when, and where do different regions of the drops and surfaces accumulate charge? Due to the challenges of performing in situ force and charge measurements with a naturally rolling drop, answers to these questions remain elusive. Nevertheless, our study comprehensively illustrates how drops interact with super liquid-repellent surfaces bearing different hydrophobic surface chemistries. It provides guidelines for developing antistatic super liquid repellency that maintains high drop mobility even under the influence of electrification.

## Experimental Section

### Synthesis of Model Stochastic Superhydrophobic Surfaces

#### Vapor-Functionalization of Soot-Templated Nanoparticles with PFOTS and DCDMS

An automated soot^26^ -deposition system is built from an array of remelted candles (Pirkka, Kesko Oyj) with wicks separated by ca. 1 cm. Six wicks created a wall of candle flame that forms the aerosolized coating mechanism. Two glass slides (Menzel-Gläser, Epredia, 75 mm × 25 mm × 1 mm) were mounted on an automated stage (OEM) with a traverse rate of ca. 1 cm s^–1^. They move over the flame wall for 18 cycles across the entire length of the slide (7.5 cm) in the *x*-axis. The flame wall is also traversed in the *y*-axis at ca. 2 cm s^–1^ to improve homogeneity of soot deposition. Coated surfaces were left to cool overnight before deposition of the silica-shell. Soot-coated surfaces were placed into the center of a desiccator (20 cm diameter, *V* = 4.2 L), where 2 mL of tetraethoxysilane (TEOS, Alfa Aesar) and 2 mL of NH_4_OH (aq, 30%, Aldrich) were each deposited on opposite sides of the perimeter edge (ca. 8 cm from the samples, and 16 cm from each other). The desiccator was then evacuated to 50 mbar and kept for 3 h to develop a silica shell. Coated surfaces were left to equilibrate overnight before calcination in a furnace (Nabertherm P330, Germany), at 600 °C (ramp: 10 °C min^–1^) for a holding time of 2 h. Templated soot surfaces were cooled in the furnace overnight, before the functionalization with either *1H*,*1H*,*2H*,*2H*-perfluorooctyltrichlorosilane (PFOTS, 97%, Sigma-Aldrich) or dichlorodimethylsilane (DCDMS, 99.5%, Sigma-Aldrich). Surfaces were placed into a desiccator (20 cm diameter, *V* = 4.2 L) at ca. 8 cm from the center, where either 0.5 or 0.2 mL of PFOTS or DCDMS were deposited respectively, at ca. 2 cm lower than the glass substrates. Silanes were deposited into the desiccator prior to evacuation to 50 mbar, for a reaction time of 3 h. This process is most optimally performed under low ambient humidity (⩽ 30%).^200^ After the reaction is completed, functionalized surfaces were then evacuated at 50 mbar (in situ without silanes present) for 30 min to remove residual silanes. DCDMS has a vapor pressure of ca. 0.2 bar. However, scanning electron micrographs of both PFOTS and DCDMS functionalized soot templates suggest that coating thicknesses are not significantly different. The primary differences are those at the nanoscale, where nanoparticles appear agglomerated and larger for DCDMS vs PFOTS.

### Dynamic Drop Contact Surface Characterization

A humidity of between 40 and 60% is used during characterization, per prior studies into surface charges and/or drop mobilities.^42,43,45,46^ The effect of charge accumulation vs humidity is well-explored,^43,57,95^ with recent work suggesting how drop slide electrification is not significantly impacted within this broad domain (<70%).^95^

#### Roll-Off Angle Analysis

Wetting was assessed through the measurement of sessile-drop based roll-off angles, by placing and averaging the rolling behavior of 5–6 drops of water (10 μL) on both PFOTS- and DCDMS- functionalized soot-templated surfaces. In these experiments, both the 30G needle depositing the drops and the stage are grounded. Substrates were grounded by exposure to high ambient water vapor.^92,96^ This is achieved by raising the local humidity *via* flooding of the stage (surrounding the substrate) with water. This allows for the probing of characteristic retention forces without the influence of charging. Dynamic images were recorded using a Biolin Attension Theta Goniometer (Finland) with a Navitar camera (1–60135, Canada). Camera settings: Exposure (7365), Gamma (2944), Gain (1521), at a magnification of 1.0 X. The CA and SA were computed by a commercially available (OneAttension) program. Data is presented as mean ± standard deviations.

To provide a detailed insight into the roll-off angle (α) of the two surface variants, we varied the tilting speed of the stage, which starts after drop placement (<2 s). This ranged from 1, 5, 15, 30, and 60 °/min. As a result, the residence time of the rolling drop during tilting can notably range from <1 s up to almost 100 s. The α analysis was performed in two ways. First, the baseline of the drop can be tracked, and once it has made discernible motion (ca. 10 pixels), the so-termed baseline roll-off angle (α_base_) can be captured. Second, we also observe the complete motion of the drop (rear-side) outside of the frame of observation (α_out_), at ca. *L* = 8 mm lateral movement. Due to the various tilt speeds, video capture rate ranged from 1.4 fps (1 °/ min) up to 14 fps (60 °/ min) to adequately match the time resolution of event capture. Characterization was performed at ca. 20–22 °C, at a relative humidity of 40–60%.

#### Dynamic Roll-Off Analysis

To assess dynamic drop rolling behaviors of water drops on both PFOTS- and DCDMS- based surfaces, a series of dynamic rolling experiments were devised. Here, the stage is pretilted at an angle of 2°, a functional roll-off angle based on α experiments. The stage is insulated with up to 1 mm thick insulation tape (NITTO, 1 in. wide). Drops are then deposited onto the surfaces while minimizing free fall, at either 0.613 μL/s or 2.5 μL/s, using a 30G needle which is grounded with a copper wire. Drops detach from the needle at ca. 6 μL. Bouncing of drops is avoided as that may introduce additional effects (Figure S13). Figure S1 These settings result in drop intervals (d*t*) of 10 and 2 s, respectively, with measurements ranging up to 47–48 drops. Location-specific contact time was ca. 0.01–0.1 s, per a rolling velocity of 10–100 mm/s. Only the data from the first 40 drops are presented. Drop volumes of ca. 4.4, 12.3, and 27.2 μL were also tested. For the sake of comparison to other techniques, a d*t* of 10 s is used in the main manuscript. In these experiments, a half-charge configuration exists, where the drop is neutral, while the surfaces gradually charge. Six repeats were performed for each surface variant, with a new location used for every test. Rolling of drops freely proceeds until they fall off the stage and is absorbed by a small piece of fibreless paper. Dynamic images were recorded using a Biolin Attension Theta Goniometer (Finland) with a Navitar camera (1–60135, Canada). Camera settings: Exposure (1683), Gamma (2496), Gain (775), at a magnification of 0.7 X. A custom light source is also employed (Raleno, 20W, 650 lx/m) to allow the capture of a contact length (*L*) up to 30 mm. Video data is then analyzed using a custom MATLAB script that tracks the rolling drops and their corresponding velocity (*v*) via center-of-mass detection. The maximum drop velocity (*v*_m_) is tracked up to rolling distances / contact lengths of 5, 10, 15, 20, and 25 mm. Data is presented as mean ± standard errors. Characterization was performed at ca. 21–22 °C, at a relative humidity of 40–60%.

#### Drop Friction Analysis (Lateral Forces)

To isolate the lateral force component of drops on the two surface variants, a custom-built drop friction apparatus (DFA, KAUST, Saudi Arabia) was used to perform drop friction-force measurements. A 10 μL drop is first deposited on a surface, which is on an insulated stage, before attaching to a polymer capillary (PMMA, 360 μm diameter). The drop is completely discharged using a grounded copper wire. An automated stage (Thorlabs, DDS220) is then used to move at a fixed velocity of 1.0 mm/s over a contact length of *L* = 10 mm before reversing on the same track. Location-specific contact time was ca. 1 s. The use of 1.0 mm/s allows for comparison to drop adhesion measurements. For the sake of comparison to other techniques, a d*t* of 10 s is used in the main manuscript. The drop interval (d*t*) of 10 s is defined as the time taken for center-to-center contact. However, at the points where the drop reverses, d*t* is considerably lower. Different drop velocities were tested (0.5–10 mm/s), but higher drop velocities led to errors and noise during analysis and are therefore excluded from this work. In these experiments, a full-charge configuration exists, where both the drop and surfaces are allowed to gradually charge. The deflection (Δ*x*) of the acrylic cantilever (flexural spring constant *k*_*x*_ = 55 mN/m) was optically imaged using a high-speed camera (Chronos 2.1 HD, 32 GB), which has a spatial resolution of 0.01 mm. This deflection is converted into absolute friction force using Hooke’s law. Characterization was performed at ca. 21–22 °C, at a relative humidity of 50–60%.

#### Drop Adhesion Analysis (Normal Forces)

To isolate the normal force component of drops on the two surface variants, a custom-built scanning drop adhesion microscope (SDAM, Aalto University, Finland) was further modified to accommodate a 10 μL water drop. We used a metal cantilever with a flexural spring constant of *k*_*y*_ = 2.74 N/m and a 3 mm 3D-printed plastic disk (ABS-like, Elegoo) to hold the drop as the force sensor. Both the cantilever and the surface/stage were electrically isolated using insulation tape (NITTO). A 10 μL water drop was placed on the disk using a Hamilton syringe with a metal needle. The drop is then completely discharged using a grounded copper wire. The automated stage moves the surface toward the drop at a fixed velocity of 1.0 mm/s, making contact, compressing to a maximum normal force *F*_push_ = 100 μN, which corresponds to the weight of a 10 μL macroscopic drop. Thereafter, the surface is retracted at a velocity of 1.0 mm/s until the drop and surface are separated. Location-specific contact time was ca. 1 s. We term this drop detachment. The maximum drop contact diameter reaches 1.3 mm while the minimum drop pinch-off diameter reaches 330 μm. Measurements were performed at a single spot with 20 sequential contact repetitions (cycles). For the sake of comparison to other techniques, a drop departure-to-contact interval (d*t*) of 10 s is used. Five repeats were performed on different locations. Surfaces are surrounded with water on uncoated parts of the substrate to assist in water vapor aided charge neutralization between experiments. The first contact serves as the reference value, where both drop and surfaces are neutralized. In these experiments, a full-charge configuration exists, where both the drop and surfaces are allowed to gradually charge. Repeat contact assesses dynamic changes in adhesion-based retention forces. Various contact velocities were tested, ranging from 0.2 to 10 mm/s. We found that higher velocities, e.g. 5 mm/s or 10 mm/s, result in errors and noise during force analysis (Figure S9). Data are presented as mean ± standard errors. Characterization was performed at ca. 21–22 °C, at a relative humidity of 40–60%.

#### From Drop Mobility Measurements to Drop Charge Characterization

To understand the observed drop rolling, friction, and adhesion behaviors, all three techniques were paired to high sensitivity electrometric live measurements (Keysight B2987A, Aalto University and Keithley 6514, KAUST). In drop rolling analysis, the rolling drop contacts a platinum wire (150 μm diameter, Pt Wire 99.99+ %) after a contact length of ca. 25 mm. The platinum wire collects the charge, delivering it to the electrometer. Each sequentially rolling drop is neutral. In drop friction analysis, a platinum wire (150 μm diameter, Pt Wire 99.99+%) is attached to a metal cantilever (Steel, 150 μm, *k*_*x*_ = 0.144 N/m), which replaces the acrylic cantilever. This allows for continuous charge measurements from the surfaces during friction measurements. In adhesion analysis, a platinum wire (150 μm diameter, Pt Wire 99.99+%) is placed directly inside the drop (on the 3D-printed disk). The nature of adhesion measurements results in periodic disconnection from the surface between contacts. Therefore, semicontinuous charge measurements occur during measurements. Data is presented as mean ± standard errors (*n* = 3–5). Paired characterizations were performed under the respective conditions present in the respective laboratories (Aalto University and KAUST).

#### Computational Details: Density Functional Theory Calculations

Quantum mechanical DFT calculations, as implemented in the CP2K software package, were employed to accurately capture the diffuse nature of electronic charge and polarization effects, both of which are critical for modeling hydrogen bond formation and physicochemical adsorption of ions. Additionally, ab initio molecular dynamics (AIMD), referred here as DFT-MD simulations were used to investigate the dynamics of system components, chemical bonding, and the stability of hydroxyl and hydronium ions at the interface. In our DFT-MD simulations, the potential energy surface is generated on the fly using DFT, which provides the ground-state energy and the corresponding interatomic forces for each time-evolved configuration. These forces are then used to propagate the nuclei according to Newton’s laws of motion. This procedure is repeated over many time steps to simulate atomic motion, typically over time scales ranging from picoseconds to nanoseconds. Further computational details are provided in the Supporting Information under “Computational Details: Density Functional Theory Calculations”.

## Supplementary Material













## Notes

Supplementary discussions on force correlation coefficient measurements, coupling charge measurements with force (rolling, friction, adhesion) measurements, differences in charge distribution behaviors for setups under insulation/discharging, correlation of charge and force measurements, and computational details of density functional theory calculations; supporting figures presenting Hirshfeld charges vs surface chemistries, radial distribution function, SEMs and AFMs, velocity-acceleration graphs, velocity-time graphs, maximum drop velocities vs contact distance/length, maximum drop velocities vs drop interval / contact distance/length, effect of discharging a friction drop, supplementary adhesion measurements, charge measurements during rolling-friction-adhesion experiments, cumulative drop charge measurements, DFT-MD simulations (perfluoroalkyl vs silicone), drop impact vs charge study, maximum drop velocities vs drop size, and DFT-MD modeling of fluoroalkyl/silicone systems (PDF)
Rolling drop (MP4)
Friction drop (MP4)
Adhesion drop (MP4)
Hydronium migration (AIMD simulations) from surfaces (MP4)
Hydroxyl migration (AIMD simulations) from surfaces (MP4)
