# Supplementary material for: Self-Accelerating Drops on Silicone-Based Super Liquid-Repellent Surfaces
Source: ACS Nano. 2025 Jun 17;19(25):23105–19. doi: 10.1021/acsnano.5c04250 (PMC12224313; doi:10.1021/acsnano.5c04250)
Supplement: Supplementary file 1 [file nn5c04250_si_001.pdf]

Supplementary Materials for

Self-Accelerating Drops on Silicone-Based Super Liquid-Repellent Surfaces

*Parham Koochak[1#], Marcus Lin[2#], Ali Afzalifar[1], Arsalan Hashemi[3], Sankara Arunachalam[2], Ayan Shoaib[2], Valtteri Turkki[1], Tapio Ala-Nissila[3,4], Dan Daniel[2], Maja Vuckovac[1], and William S. Y. Wong[1]**

Department of Applied Physics, School of Science, Aalto University, FI-02150 Espoo, Finland[1]

Division of Physical Sciences and Engineering, King Abdullah University of Science and Technology (KAUST), Thuwal 23955-6900, Saudi Arabia[2]

MSP Group, Quantum Technology Finland Center of Excellence, Department of Applied Physics, Aalto University, FI-00076 Espoo, Finland[3]

Interdisciplinary Centre for Mathematical Modelling and Department of Mathematical Sciences, Loughborough University, Loughborough, Leicestershire LE11 3TU, United Kingdom[4]

Keywords: Drop rolling, Drop friction, Drop adhesion, Drop electrification, Charge Suppression

[#]Equal contributions
*Corresponding author: william.wong@aalto.fi

This file includes:

Supplementary Discussion

- *Force Correlation Coefficient Measurements by Fitting with 1st-order kinetics.*
- *Coupling Charge Measurements with Force (Rolling, Friction, Adhesion) Measurements.*
- *Differences in Charge Distribution Behaviors for Insulated vs. Discharged Setups.*
- *Correlating Charge and Force Measurements (DCDMS-based Silicone).*

Supporting Figures S1-15

Supporting Tables S1-4

Computational Details: Density Functional Theory Calculations

- *Radial distribution function (RDF).*
- *Partial charge analysis – Hirshfeld.*

Supporting Movies M1-5

- 1) Rolling drop on perfluoroalkyl (PFOTS) *vs.* silicone (DCDMS) based surfaces.
- 2) Friction drop on perfluoroalkyl (PFOTS) *vs.* silicone (DCDMS) based surfaces.
- 3) Adhesion drop on perfluoroalkyl (PFOTS) *vs.* silicone (DCDMS) based surfaces.
- 4) Hydronium Migration (DFT-MD Simulations) from surfaces.
- 5) Hydroxyl Migration (DFT-MD Simulations) from surfaces.

Supplementary Discussion

Force Correlation Coefficient Measurements by Fitting to 1st-order Kinetics

Forces measured on surface variants are presented in both absolute and dimensionless forms (**Table S1**). The initial (i = 1) and equilibrium states ($i \approx \infty$) are derived by fitting to 1st-order kinetics. To identify characteristic trends, we introduce the force correlation coefficient (F_∞ / F_1), which represents the net change in forces acting on drops across the three methods used.

Perfluoroalkyl-based surfaces exhibit no discernible trends in rolling drop behaviors, making them unsuitable for dynamic analysis. The discussion of perfluoroalkyl (PFOTS) surfaces is limited to the role of a control benchmark for silicone (DCDMS) surfaces. Notably, friction measurements on both surface variants deviated significantly from rolling and adhesion measurements, as forces consistently increase with continuous contact on both perfluoroalkyl-based and silicone-based surfaces. This suggests that friction measurements may not accurately reflect rolling behaviors. Consequently, based on the trends observed in **Table S1**, an extensive discussion of the friction method with regards to charge-to-mobility correlations is excluded from the manuscript. Future work could explore how friction measurements are influenced by charge-induced effects; however, this investigation lies beyond the scope of the current study.

Table S1. Forces (Absolute, Dimensionless, and Coefficients): Initial ($i0$) vs. Equilibrium (∞)

	PFOTS			DCDMS		
**Force Method ($F_{r,i}$)**	F_1	F_∞	F_∞/F_{i0}	F_1	F_∞	F_∞/F_{i0}
Rolling, $F_{r,i}$ (μN)	0.76	0.77	1.01	1.16	0.99	0.85
Rolling, $F_{r,i}/\gamma D$ (-)	4.7x10^{-3}	4.7x10^{-3}		7.1x10^{-3}	6.1x10^{-3}	
Friction, $F_{fric,i}$ (μN)	0.97	35	36.1	3.07	9.33	3.04
Friction, $F_{fric,i}/\gamma D$ (-)	5.04x10^{-3}	182x10^{-3}		16x10^{-3}	48x10^{-3}	
Adhesion, $F_{adh,i}$ (μN)	5.58	5.39	0.97	13.6	7.09	0.52
Adhesion, $F_{adh,i}/\gamma D$ (-)	29x10^{-3}	28x10^{-3}		71x10^{-3}	37x10^{-3}	

Coupling of Charge Measurements with Rolling, Friction, and Adhesion Measurements

Without charge measurements, key differences already exist between the rolling, friction, and adhesion analysis. For the ease of reference, key properties are included in **Table S2**.

Table S2. Rolling, Friction, and Adhesion Measurements (w/o charge measurements).

**Characteristics**	**Rolling**	**Friction**	**Adhesion**
Driving Force	Gravity	Mechanical Input	Pressing Load
Vector (to Surface)	Normal and Lateral	Lateral	Normal
Velocity	Surface driven	User-defined	User-defined
Drop	Pristine	Re-Used	Re-Used

<u>*Rolling:*</u> In rolling drop measurements, the charge apparatus is decoupled from the assessment of rolling, allowing undisturbed charge measurements as drops leave the surface. From a charge distribution perspective, each new drop begins in a neutral state, but test surfaces will experience a progressive accumulation of charges over time. Drops that roll across the surface then charge up as a consequence of how the test surfaces are charged. This accumulation or suppression of charges between drops and surfaces induce mobility variations which are observed through changes in drop velocity (Figure S10c-d).

Rolling drop analysis (mobility and charge) contrasts with both friction and adhesion measurements. The coupling of charge measurements in these latter instances require invasive probing of force sensing drops with a platinum wire, which can influence force measurements. Key differences are highlighted below:

<u>*Friction:*</u> In friction measurements, a platinum wire continuously drains charges from a slow-moving drop (U_x = 1 mm/s). This prevents significant charge accumulation. At timescales that govern flow of charges (likely *ca.* milliseconds),[1] the drop remains essentially neutral. However, as the drop cycles along the same track, the track itself is directly connected to the platinum wire, resulting in a continuously discharged surface. As a result, friction forces remain

[1] The velocity of electromagnetic waves through good conductors is defined by $v = 0.41\sqrt{\frac{f}{\sigma_r \mu_r}}$. where f is the frequency (Hz), σ_r is the conductivity of the material (*S/m*), and μ_r is the magnetic permeability of the material (*H/m*).

completely unaffected during cyclic friction tests. This behavior contrasts strongly with measurements performed with insulated drops (without discharge / measurements) but clearly demonstrates how surface and drop charge accumulation likely causes increase in friction forces. For both surface variants, the so-called characteristic retention force displays very similar values (Figure S10c-d). Speculatively, this suggests that residual surface charge could have more influence on characteristic retention forces that has so far only been primarily attributed to surface chemistry. However, as surface chemistry and charge are inter-dependent (see DFT-MD analysis), this may not be such an unexpected outcome.

Adhesion: In adhesion measurements, a platinum wire continuously grounds the drop, maintaining its neutrality. During the slow contact-and-detachment process (U_y = 1 mm/s), rapid drop detachment induces charge separation. The drop becomes spontaneously positively charged while the surface becomes negatively charged. However, the surface is not discharged since it is now disconnected from the drop. Over the next few milliseconds, the drop becomes neutralized by the platinum wire as charges are collected by the measurement device. In adhesion measurements, the detachment process induces charge separation much more rapidly than in friction measurements. This allows the drop to momentarily experience variations in adhesion forces due to electrification prior to being discharged through the wire. As a result, we still observe (Figure S10e-f) how silicone-based surfaces experience suppression of drop electrification and adhesion forces, albeit with noise due to diminished charge-induced force sensitivity.

Differences in Charge Distribution Behaviors in Insulated vs. Discharged Setups

To highlight key differences (or lack thereof) between insulated and discharged setups, we collated the primary attributes of each measurement type in **Table S3** and **Table S4**. We observed clearly how a momentarily neutral drop (fresh or actively discharged) is able to detect charge-induced forces if the test surface itself accumulates charge. Rolling and adhesion measurements show similar force trends for both surface variants (**Table S3-4**) even under insulated/discharged conditions but completely contrasted friction measurements. Under friction measurements, forces always build-up if either (1) the surface or (2) both the surface and the drop are charged. This occurs immediately upon contact motion of drops. There are no friction force variations if both surfaces and drops are actively neutralized (**Table S3** and **Table S4**).

Table S3. Insulated *vs.* Discharged Setups for Perfluoroalkyl-Based Surfaces

**Method**	**Insulated (Force Measurements Only)**			**Discharged (Force-Charge Measurements)**		
**Component**	$Drop_{initial}$	Surface	Force	$Drop_{initial}$	Surface	Force
Rolling	Neutral	Charged	No change	Neutral	Charged	No change
Friction	Charged	Charged	Increase	Neutral	Neutral	No change
Adhesion	Charged	Charged	No change	Neutral	Charged	No change

Table S4. Insulated *vs.* Discharged Setups for Silicone-Based Surfaces

**Method**	**Insulated (Force Measurements Only)**			**Discharged (Force-Charge Measurements)**		
**Component**	$Drop_{initial}$	Surface	Force	$Drop_{initial}$	Surface	Force
Rolling	Neutral	Charged	Decrease	Neutral	Charged	Decrease
Friction	Charged	Charged	Increase	Neutral	Neutral	No change
Adhesion	Charged	Charged	Decrease	Neutral	Charged	Decrease

Correlating Charge and Force Measurements (DCDMS-based Silicone)

We first define the decay in normalized force or charge as,

$\hat{y}_{\mathrm{i}} = Ae^{-Bi} + C$, where $\hat{y}_{\mathrm{i}} = \hat{F}_{\mathrm{i}}$ or $\hat{Q}_{\mathrm{i}}$. (S1)

Two boundary conditions exist, and we revise the "start" of i from 1 (count) to 0 (native):

1) $i = \infty$, $\hat{y}_{\mathrm{i}} = C = \hat{y}_{\infty}$. [The equilibrium force ($\hat{F}_{\infty}$) or charge ($\hat{Q}_{\infty}$)].
2) $i = 0$, $\hat{y}_{\mathrm{i}} = A + \hat{y}_{\infty} = 1$. Therefore, $A = 1 - \hat{y}_{\infty}$. [The magnitude of change]

Therefore, Equation S1 becomes,

$$\hat{y}_{\mathrm{i}} = (1 - \hat{y}_{\infty})e^{-Bi} + \hat{y}_{\infty} \quad \text{(S2)}$$

B is attributed to drop contact frequency, which is ultimately impacted by drop interaction history (t) and surface time constant (τ). However, the correlated force and charge measurements are performed in a way where they possess (*e.g.* rolling *vs.* adhesion) slightly different t, despite keeping "event interval" dt constant. Therefore, we conservatively correlate the native drop interaction number (i - 1), while terming the pre-factor as a time-dependent discharge / force decay parameter (λ). We rewrite Equation S1 as

$$\hat{y}_{\mathrm{i}} = (1 - \hat{y}_{\infty})e^{-\lambda(i-1)} + \hat{y}_{\infty} \quad \text{(S3)}$$

where $\lambda(i-1) = f(t, \tau)$.

We use λ, $\hat{y}_{\infty}$ and cross model fitting (R^2) to assess similarities or differences in the decay of force and charge.

Supporting Figures

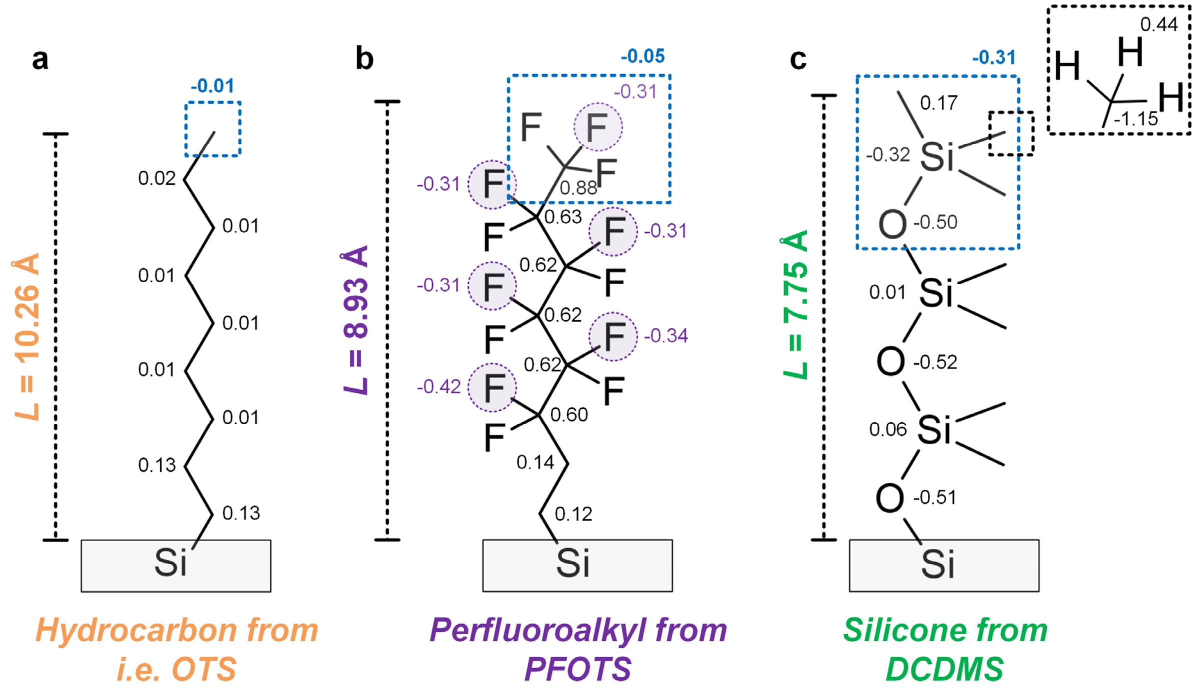

Figure S1. Hirshfeld Charges on Atoms of (a) Alkyl-chain, (b) Perfluoroalkyl-chain, and (c) Dimethyl-silicone chain. The average length of each species is indicated at the corresponding molecular size, taken from 10 snapshots of DFT-MD calculations. In (a) and (c) respectively, the hydrogen (H) and methyl ($-CH_3$) groups are hidden. The collective charge is depicted on the central atom (*i.e.* C, Si, O). The blue box highlights the terminal charge of the molecular chains, with (a) and (b) represented by terminal carbon atom as the methylene ($-CH_2$) and difluoromethylene ($-CF_2$) groups are almost neutral. (c) is represented by the terminal unit, as part of the repeat unit, $Si(CH_3)_nO$. The terminal methyl unit and its partial charges are represented in the dashed box.

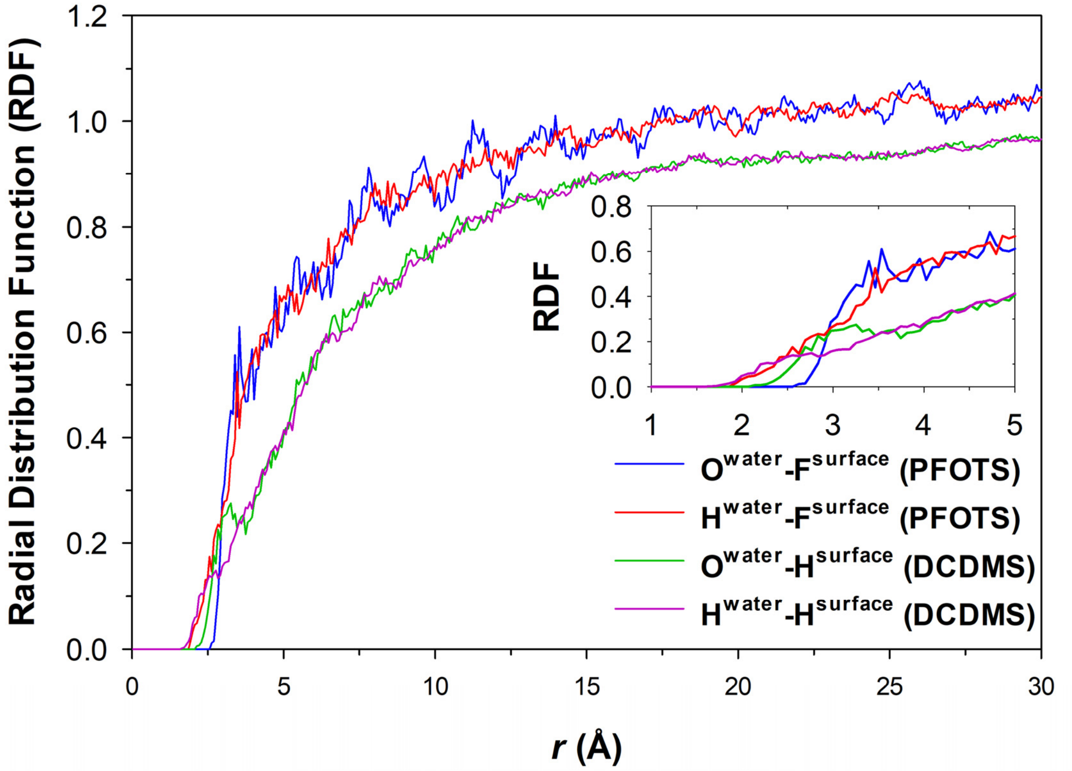

Figure S2. The Radial Distribution Function (RDF) for Gauging Proximity of Water-to-Surface, Representing a Prediction of Hydrophobicity. In this instance, water's hydrogen (H^{water}) and oxygen (O^{water}) are shown with respect to surface elements, specifically fluorine atoms ($F^{surface}$) in PFOTS-based perfluoroalkyls and hydrogen atoms ($H^{surface}$) in DCDMS-based silicones.

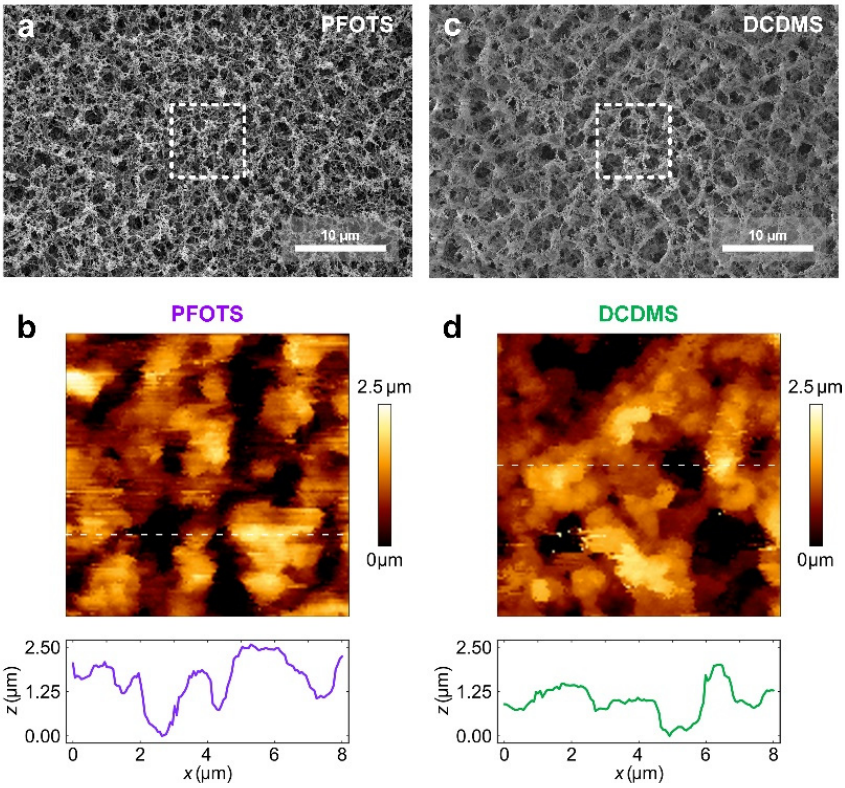

Figure S3. Surface Profile Analysis of (a-b) PFOTS- and (c-d) DCDMS- functionalized soot-templated silica nanoparticles. Scanning electron microscopy analysis (30 x 45 µm^2) shows how surface morphologies between (a) PFOTS (R_q = 461 nm) and (c) DCDMS (R_q = 437 nm) functionalized surfaces are similar in profile, with networked-like interconnected particles having deeper micrometric grooves. Atomic force microscopy analysis (8 x 8 µm^2) corroborates these observations, therein showing very similar nano- and micro-scale topographical roughness.

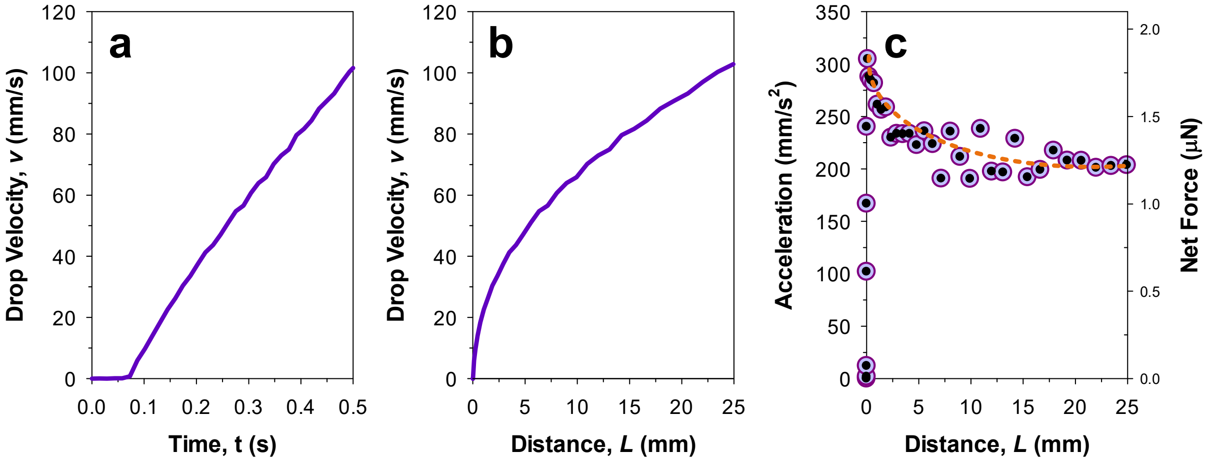

Figure S4. Velocity *vs.* Time / Distance (Contact Length), and Acceleration/Force *vs.* Distance (Contact Length): Reference Figure 3c. (a) Velocity *vs.* time analysis, (b) velocity *vs.* distance / contact length analysis, and (c) acceleration / force vs. distance / contact length analysis. Acceleration is *ca.* 224 mm/s^2 for the entire contact length, if starting at 0.06 s and *ca.* 205 mm/s^2 and only using acceleration from final 0.06 s. This represents an 8% error. In this work, we average across the entire contact length, with repeat experiments (n = 6) giving a value of *ca.* 216 mm/s^2.

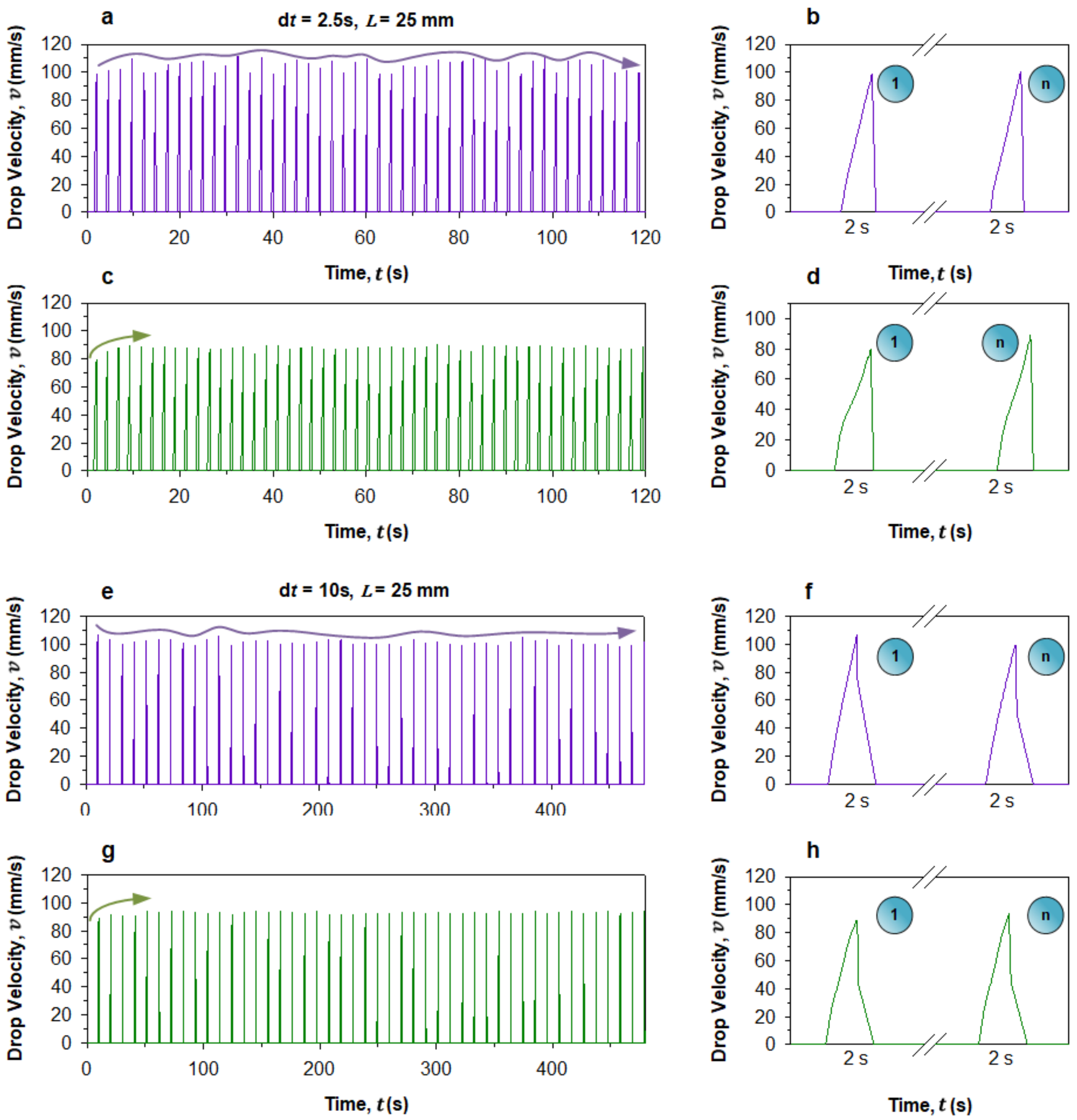

Figure S5. Live Velocity Measurements of Rolling Drops. Variable time intervals were tested at over (a-d) dt = 2.5 s and (e-h) dt = 10 s. (a-b, e-f) Drops on perfluoroalkyl-based surfaces show sporadic inter-drop velocities compared to (g-h) silicone-based surfaces. Acceleration of drops take place in a largely linear manner and does not reach plateaus. This allows for the extraction of velocity, acceleration, and the retention forces experienced by sequential drops.

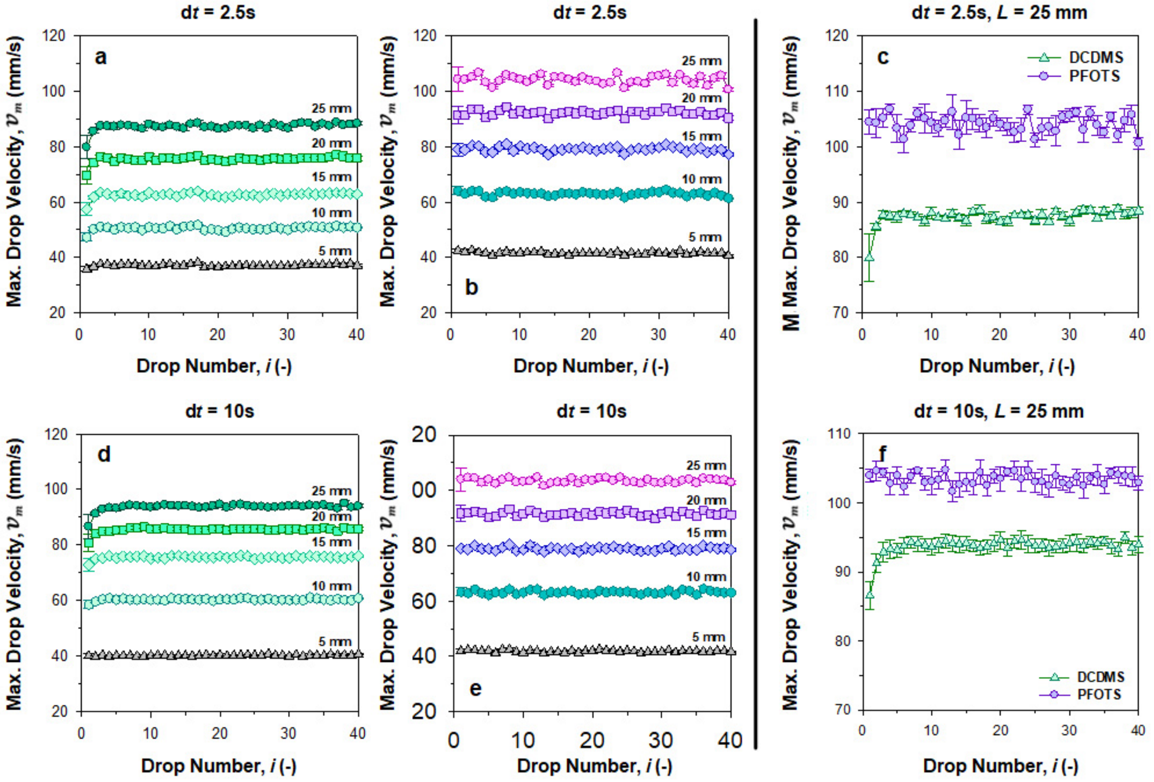

Figure S6. Maximum Drop Velocities (n = 6, mean ± standard errors). Peak velocities for (a,d) silicone-based and (b,e) perfluoroalkyl-based surfaces are extracted and presented in terms of the contact length, L. Variation in drop interval (dt) at (a-c) 2.5 s and (d-f) 10 s does not significantly impact dynamic trends beyond marginal changes in drop velocities. Magnified versions of perfluoroalkyl- *vs.* silicone- based surfaces at L = 25 mm are included in (c) dt = 2.5 s and (f) dt = 10 s for reference.

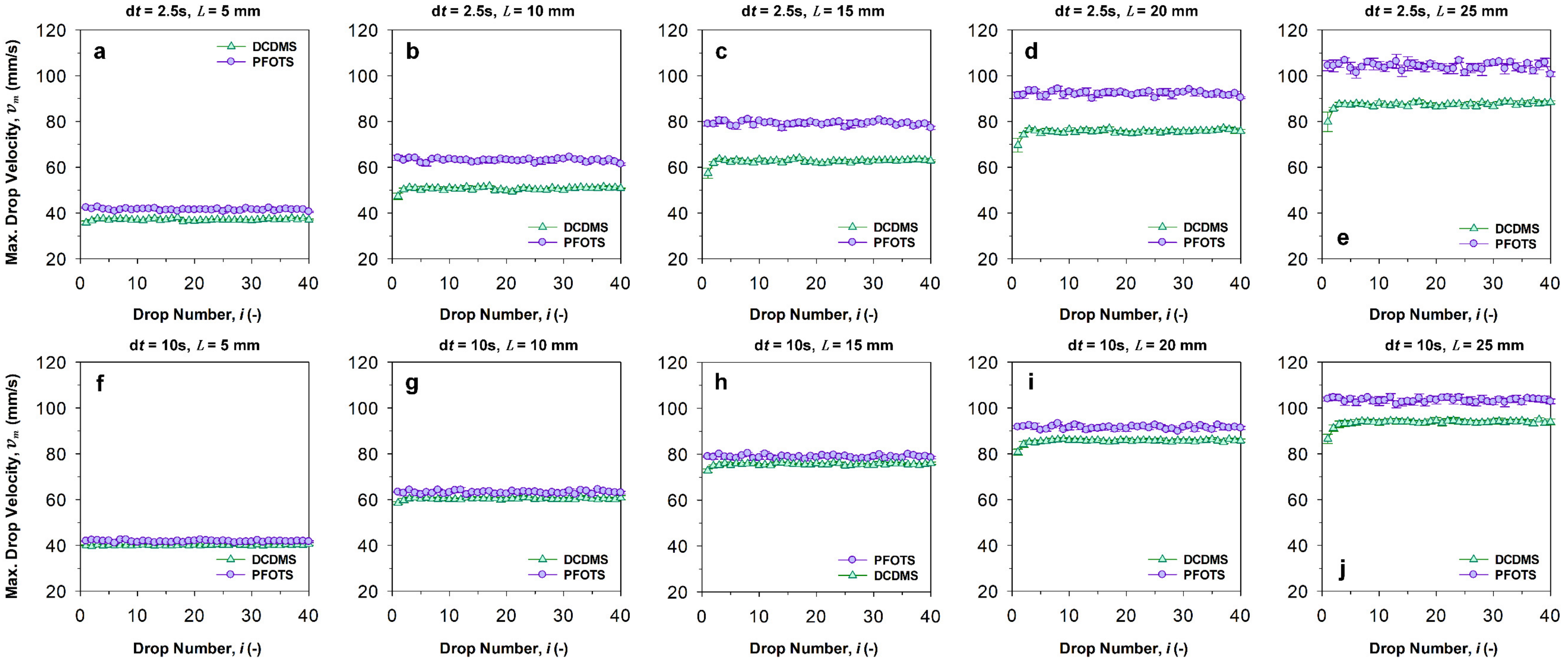

Figure S7. Maximum Drop Velocity Plots w.r.t. Drop Interval, dt and Contact Length, L. At (a-e) dt = 2.5 s *vs.* (f-j) dt = 10 s, rolling drops on perfluoroalkyl surfaces (purple data) appear to reach the same final velocity of *ca.* 105 mm/s. However, silicone surfaces (green data) appear to start and end at a slower velocity. Additionally, the dynamics of acceleration appears to be stronger when dt = 2.5 s. In this work, we examine the possibility where this is linked to the effects of surface charge, which is known to possesses time-dependent relaxation behaviors. Overall trends hold across both sets of experiments, and for the sake of conciseness, dt = 10 s is used to focus the discussion within the manuscript. n = 6, mean ± standard errors.

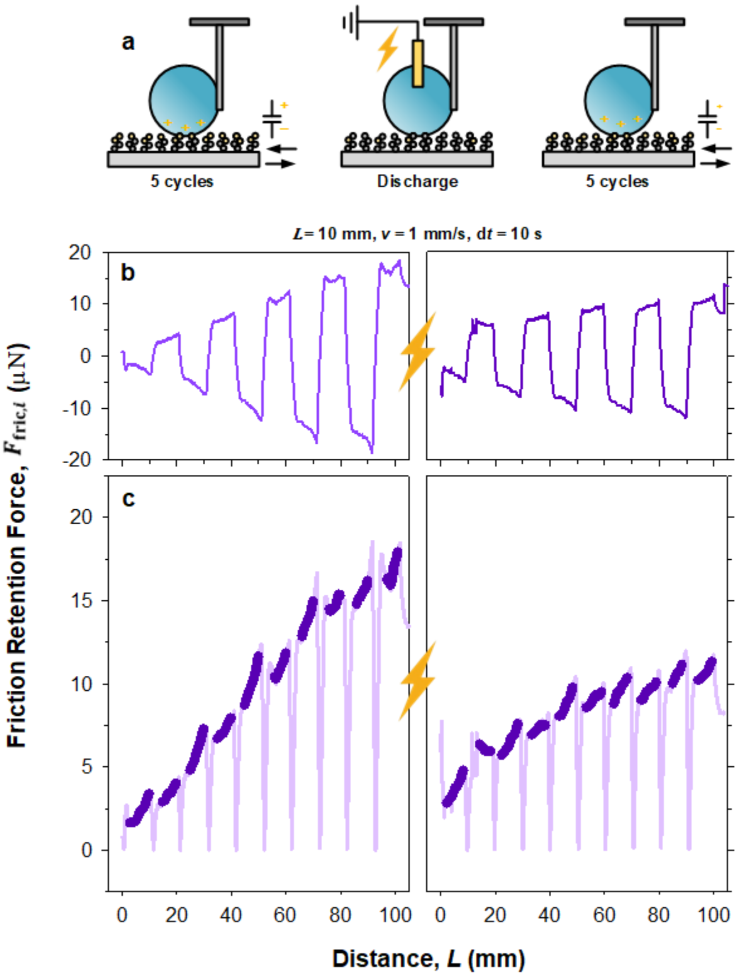

Figure S8. Charging-attributed Mobility Variations in Friction Measurements. (a) Charge-discharge of friction drops between (b-c) 10 running cycles show how the friction retention force, F_{fric}, returns to a lower value upon electrical discharge.

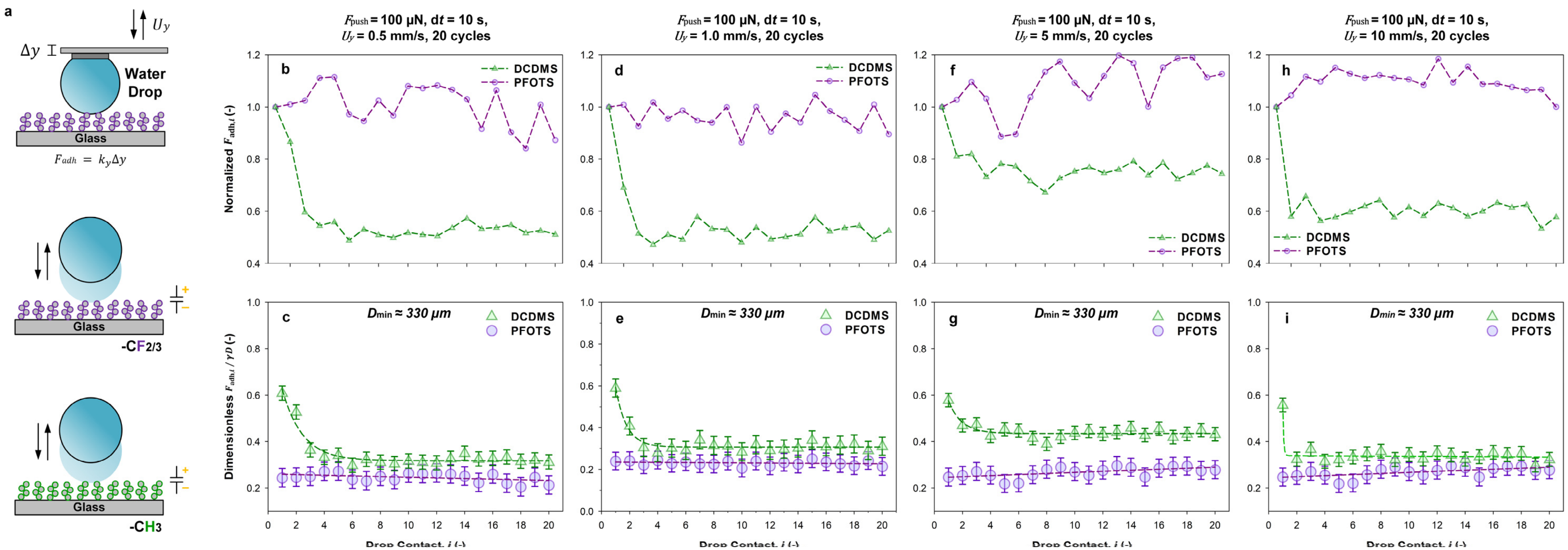

Figure S9. Detaching Drops on Perfluoroalkyl- and Silicone- based Surfaces under Different Contact Velocities in Adhesion Measurements. (a) 10 µL drops are made to contact surfaces at fixed velocity in the y-axis, U_y, with (b-c) at 0.5 mm/s, (d-e) at 1.0 mm/s, (f-g) at 5 mm/s, and (h-i) at 10 mm/s. Both the (b, d, f, h) normalized and (c, e, g, i) dimensionless forces are reported. Drops contact surfaces under a D_{max} of up to 1.3 mm during press-in contact and D_{min} of *ca.* 330 µm during pull-off. Despite higher noise at higher contact velocities ($U_y \geq 5$ mm/s), overall trends hold: perfluoroalkyl-based surfaces hold a steady force while silicone-based surfaces experience continuously lowered adhesion forces. Therefore, to preserve conciseness of the manuscript, only U_y at 1.0 mm/s is reported. n = 5, with mean ± standard error.

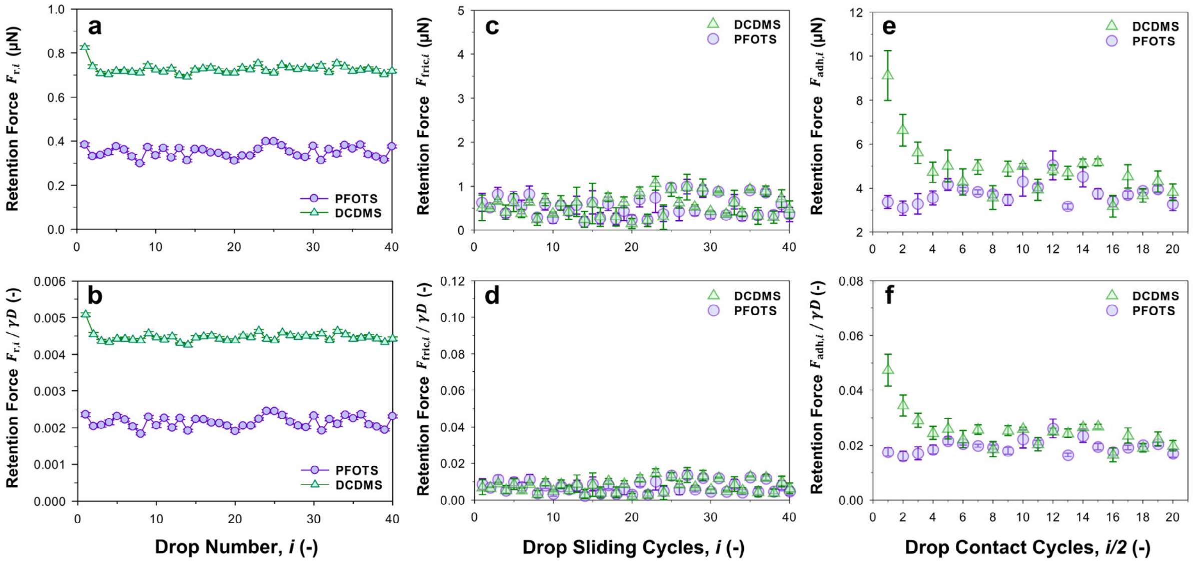

Figure S10. Drop Rolling *vs.* Drop Friction *vs.* Drop Adhesion on Perfluoroalkyl- and Silicone- based Surfaces during Charge Measurements. Measurements are paired to Figure 5 in the main manuscript. (a-b) Rolling-based retention force trends: Steady and dropping retention forces on perfluoroalkyl- and silicone- based surfaces, respectively. (c-d) Friction-based retention force trends: discharged perfluoroalkyl- and silicone- based surfaces no longer show friction-based retention force variations. This indicates that the time acted on by friction forces are larger than the time taken to accumulate charges. (e-f) Adhesion-based retention force trends: Steady and dropping retention forces on perfluoroalkyl- and silicone- based surfaces, respectively. n = 3-5, with mean ± standard error.

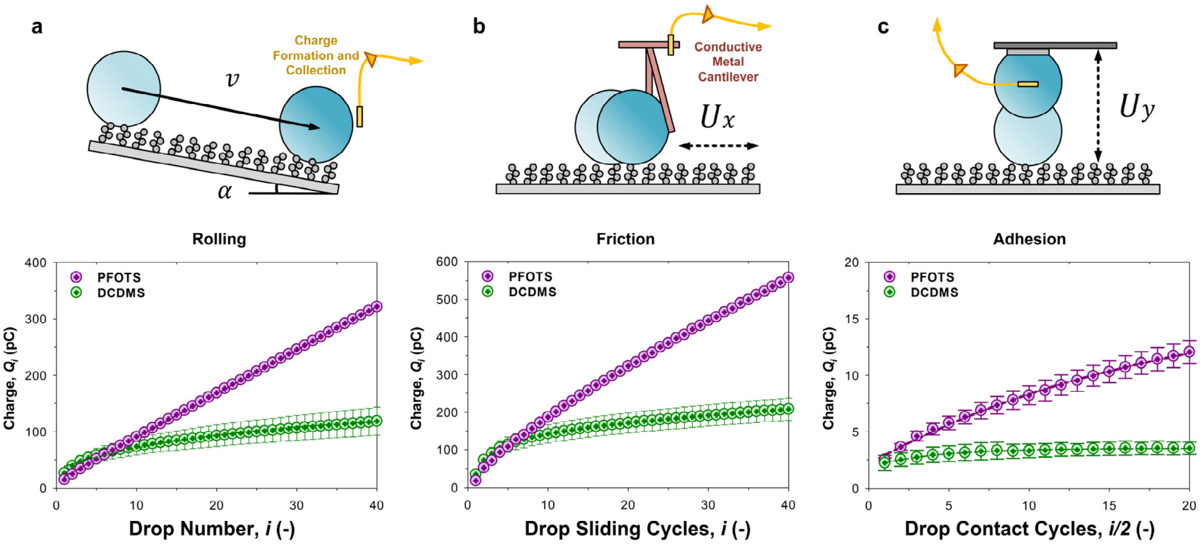

Figure S11. Cumulative Drop Charge Measurements from (a) Rolling-, (b) Friction-, and (c) Adhesion- based Drop Mobility Measurements. All charge measurements were performed (semi)continuously alongside force measurements, representing the cumulative reverse charge (*e.g.* 100 pC cumulative drop charge = -100 pC cumulative surface charge) on surfaces, assuming minimal discharge over experimental runtime. n = 3-5, with mean ± standard error.

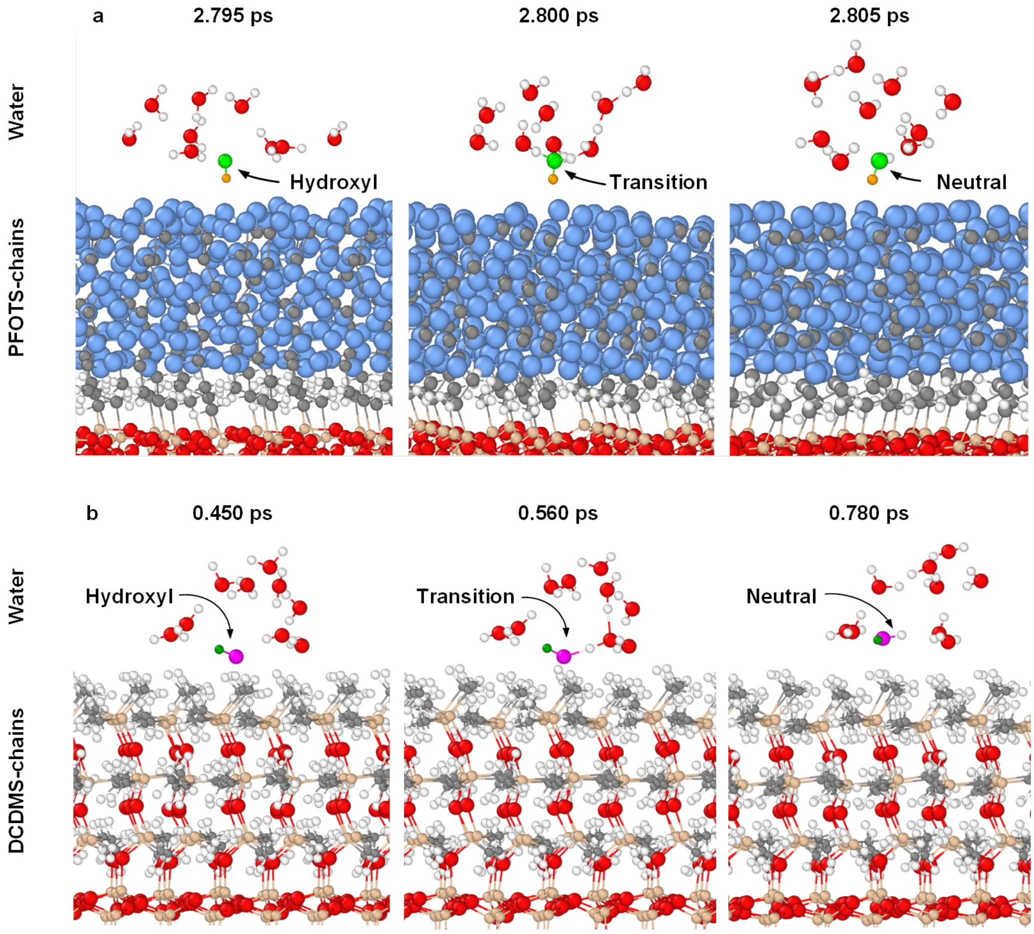

Figure S12. DFT-MD Simulations: Structure of hydroxyl and water molecules near the (a) PFOTS- and (b) DCDMS-grafted surfaces. Hydroxyls are adsorbed at the interfaces for finite time before transiently forming an O-H bond that culminates in water formation. Red, white, cyan, gray, and orange spheres represent O, H, F, C, and Si atoms, respectively. The oxygen and hydrogen atoms of the hydroxyl ion are highlighted in green/orange (PFOTS) and pink/dark-green (DCDMS) for clarity. For better visualization, extra water molecules were removed from the MD snapshots.

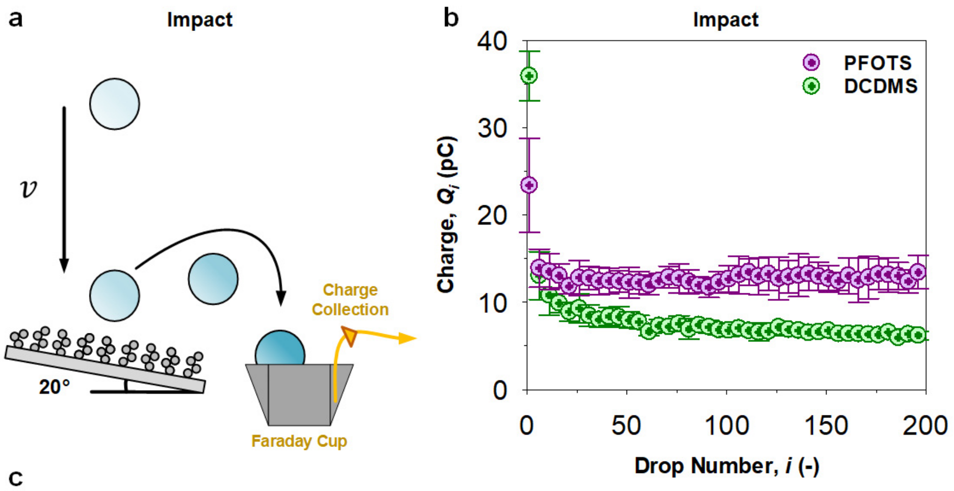

c

Surfaces:	PFOTS			DCDMS		
Charge Method (Q_i)	$Q_{\max}$	Q_∞	$Q_\infty/Q_{\max}$	$Q_{\max}$	Q_∞	$Q_\infty/Q_{\max}$
Rolling, $Q_{r,i}$ (pC)	15 ± 0.6	7.5 ± 0.2	**0.50**	26 ± 5	1.5 ± 0.5	**0.06**
Friction, $Q_{fric,i}$ (pC)	37 ± 2	12 ± 1	**0.33**	35 ± 14	1.6 ± 0.6	**0.05**
Adhesion, $Q_{adh,i}$ (pC)	2.5 ± 0.2	0.34 ± 0.03	**0.14**	2.2 ± 0.7	0.01 ± 0.03	**0.005**
Drop Impact, $Q_{di,i}$ (pC)	23 ± 5	13 ± 2	**0.57**	36 ± 3	6 ± 1	**0.16**

Figure S13. Drop Impact Study: From Bouncing to Charging. (a) Sequential drops (200 to reach quasi-equilibrium) were bounced off surfaces into a Faraday cup for (b) charge collection.[1] Drop detachment height ≈ 1 cm. Drop volume ≈ 6.6 µL. Drop velocity ≈ 0.32 m/s. We ≈ 3.52. Surface tilt ≈ 20°, relative humidity = 60%, temperature = 19 °C, n = 3. (c) Drop charge suppression using an impacting-bouncing drop is still evident between PFOTS *vs.* DCDMS. However, with impacting-bouncing drops, DCDMS-based surfaces experience the highest charge correlation coefficient among all 4 methods. Speculatively, this is attributed to the much higher drop retraction velocity and how drops may have penetrated deeper into the nano/micro-structures during impact.

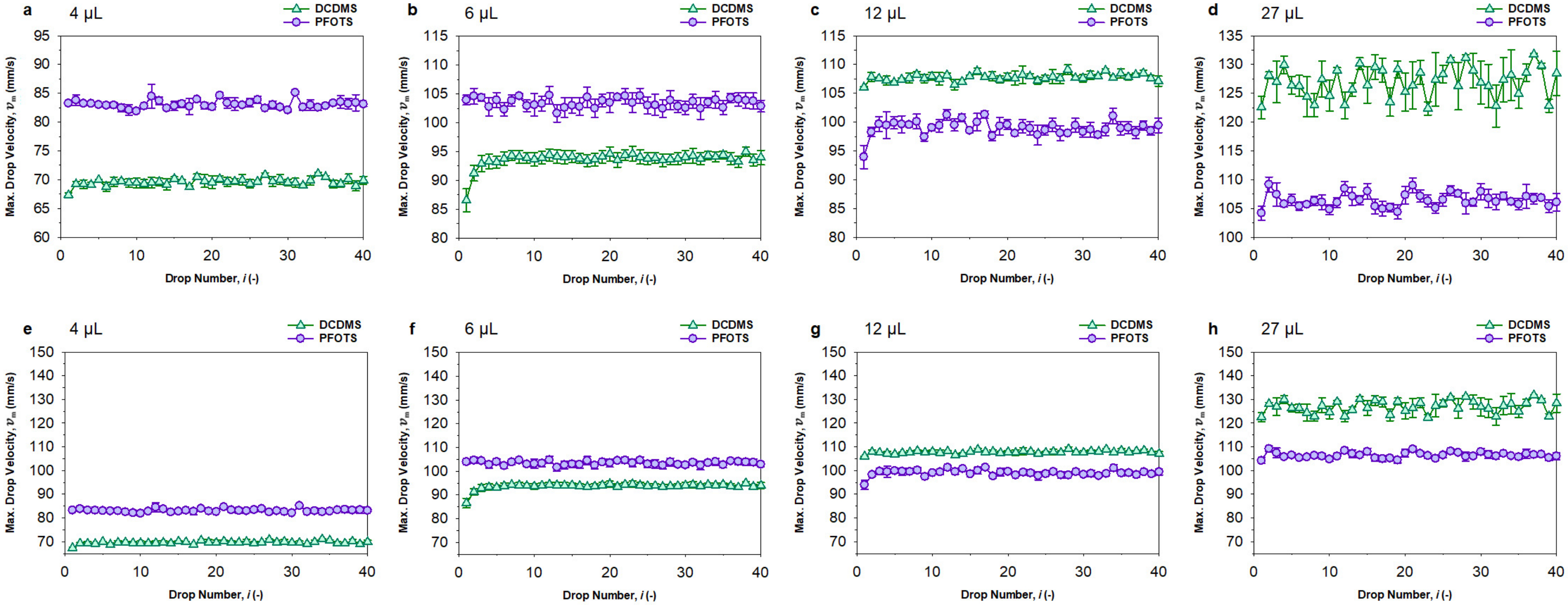

Figure S14. Drop Size Variation – Rolling Drop Measurements. Drops of various sizes (a, e: 4 µL, c, g: 12 µL, and d, h: 27 µL) and from the main manuscript (b, f: 6 µL) were dispensed using flat-tipped stainless-steel needles at the respective gauge sizes of 23G, 16G, and 31G. Top row (a, b, c, d): y-axis range: 35 units (mm/s), magnified for analysis. Bottom row (e, f, g, h): y-axis range: Uniform from 65-150 mm/s for comparison between datasets. Needles were grounded. Drops detach naturally from needles and were tracked for *ca.* 25 mm. Due to the non-linear and opposing contributions between gravitational force, $mgsin(\alpha)$ and surface area induced charge-induced retention forces (F_e), different drop sizes can result in non-linear observations. As drop size is increased, from (a, e) 4 µL to (b, f) 27 µL for silicone-based surfaces, observations of self-acceleration between drops first increases and then gradually diminishes. Curiously, the same appears to occur for (b, f) 4 µL to (c, g) 27 µL on perfluoroalkyl-based surfaces, albeit at a different drop size. This potentially indicates the universality of charge-suppression induced self-acceleration of rolling drops. Nonetheless, the charge suppression behavior of silicone-based surfaces appears to manifest in the maximum rolling velocities *vs.* drop size. Drops on silicone-based surfaces exceeded the maximum rolling velocity of perfluoroalkyl-based surfaces at 12 µL and significantly exceeded them at 27 µL. In contrast to this, the maximum rolling velocity of drops on perfluoroalkyl-based surfaces stagnated at *ca.* 100 ± 5 mm/s even with larger drop volumes (6 µL to 27µL), depicting negligible differences between experimental sets. We currently attribute this to the charge-induced withholding forces that a perfluoroalkyl-based surface experiences. For a silicone-based surface, these forces rapidly dissipate even upon limited drop contact, which are (likely) positively correlated to larger drop volumes / contact area.

Computational Details: Density Functional Theory Calculations

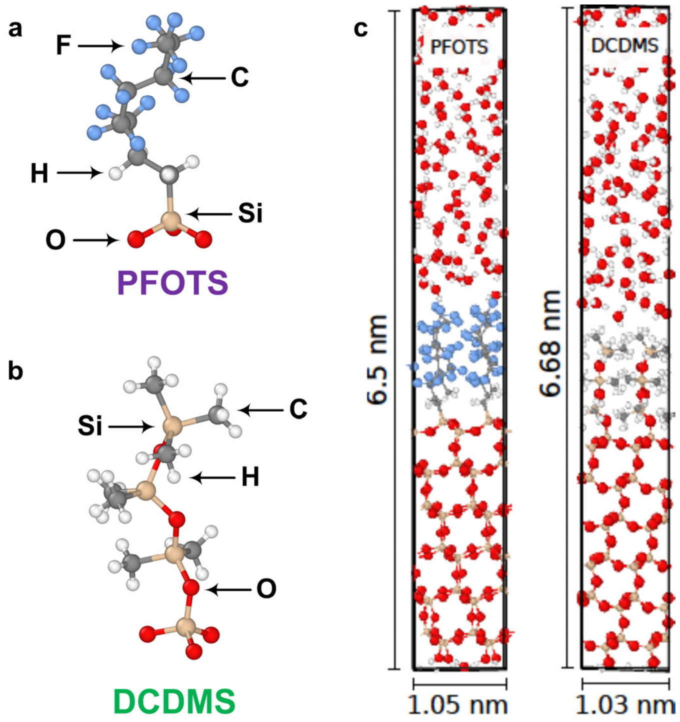

Figure S15. Model systems for (a) PFOTS and (b) DCDMS grafted surfaces interacting with water molecules. Red, white, cyan, orange, and gray spheres represent oxygen, hydrogen, fluorine, silicon, and carbon atoms, respectively.

The electronic structure calculations were performed using quantum mechanical density functional theory[2] (DFT) with the Gaussian plane-wave (GPW) method, as implemented in CP2K[3]. CP2K distinguishes itself through the integration of localized Gaussian basis sets with plane-wave techniques, offering a versatile framework for modeling a broad spectrum of systems, including molecules, liquids, solids, and their interfaces. The plane-wave and Gaussian basis sets were truncated at energy cutoffs of (i) 400 Ry and 40 Ry for *ab initio* molecular dynamics (AIMD, termed here DFT-MD) simulations, and (ii) 700 Ry and 70 Ry for energy minimization and partial charge analysis calculations. Self-consistent iterations were converged when the energy deviation was below 10^{-6} Ha for (i) and 10^{-8} Ha for (ii). The Perdew-Burke-Ernzerhof (PBE) exchange-correlation functional[4] was used throughout this work. Valence electrons were treated with the optimized DZVP-MOLOPT-SR-GTH Gaussian basis sets,[5] while norm-conserving GTH-PBE pseudopotentials[6] were employed for ionic cores. To

account for dispersion interactions, DFT-D3 van der Waals (vdW) corrections with Becke-Johnson damping were applied.[7, 8] All calculations included spin polarization. For periodic systems, DFT-MD simulations were conducted in the *NVT* ensemble for 25 ps with a time step of 0.5 fs. Temperature was maintained at 300 K using the velocity rescaling thermostat by Bussi et al.[9]

To ensure consistency with experiments, the (111) surface of cubic SiO_2 was modeled, with O-terminated surface passivated by hydrogen. The silica slab was about 2.5 nm in height and contained 144 atoms, with an additional 4 hydrogen atoms upon passivation. The hexagonal supercell had an out-of-plane lattice constant of 6.5 nm, creating a vacuum region of approximately 4 nm, which was later filled with the grafting molecules and water. At this stage, four similarly lengthed perfluoroalkyl and dimethyl-silicone molecules were separately grafted onto the Si-terminated surfaces in a stepwise manner to ensure system stability. The perfluoroalkyl is represented entirely by the post-reacted PFOTS molecule, comprising of a chain of two CH_2 groups, followed by five CF_2 groups, and terminating with a CF_3 group, arranged from the SiO_2 surface toward the water interface (Figure S15a). The dimethyl-silicone consists of three repeat units of post-reacted DCDMS, as $Si(CH_3)_nO$, where $n = 2$ for the two units closest to the SiO_2 surface and $n = 3$ for the outermost unit, which interacts with water (Figure S15b). In the following discussion, we term these the perfluoroalkyl- and dimethyl-silicone grafted surfaces, respectively. After geometry optimization, the in-plane lattice constants were set to 1.05 nm for the dimethyl-silicone chain and 1.03 nm for the perfluoroalkyl chain. A total of 90 water molecules were randomly distributed within the vacuum region of the optimized molecularly grafted surface simulation box using Packmol.[10] With the in-plane lattice vectors fixed, the water-solvated system was re-optimized to allow relaxation along the *z*-axis. To account for system expansion at room temperature, an additional 0.1 nm was added to the *z*-axis length after optimization. The final *z*-lengths were set to 6.50 nm for perfluoroalkyl

and 6.68 nm for dimethyl-silicone in the water-included calculations (Figure S15c). The configurations were visualized and analyzed, as needed, using the OVITO[11] package.

To model hydroxyl (OH^-) group interactions with the surface and compute their lifetime, two different systems were set up for each molecularly grafted surface. The final structures obtained from the pristine water system were used as the initial configurations, with the reaction ($2H_2O \rightleftharpoons H_3O^+ + OH^-$) implemented. In the first scenario, the OH^- group was placed near the surface, while the H_3O^+ ion was positioned 3 nm away. Such a model is still charge-neutral. In the second scenario, the positions of these species were switched. The interactions of radical species were monitored over 25 ps of DFT-MD simulations.

Radial distribution function (RDF)

We find that the radial distribution function (RDF, Figure S2) of water oxygen atoms with respect to the surface in PFOTS begins to increase approximately 1 Å higher than that of water hydrogen atoms. Considering the water OH bond length of 0.96 Å, this corresponds to a contact angle of about 90°, whereas in the DCDMS system, the oxygen RDF starts rising about 0.6 Å higher than hydrogen, resulting in a contact angle of about 37°. Since the contact angle quantifies hydrophilicity and hydrophobicity,[12, 13] this finding indicates that DCDMS is more hydrophilic than PFOTS, where water OH groups tend to orient vertically. Furthermore, water maximizes H-bonding with the surface in regions exhibiting local hydrophilicity.[13, 14] Comparing the RDFs of the two systems, we observe that water hydrogen atoms, on average, are positioned ~ 2.5 pm closer to the surface in the case of DCDMS compared to PFOTS.

Partial charge analysis - Hirshfeld

Hirshfeld charges were computed (Figure S1) to show electronic properties of three classical chemistry configurations: 1) alkyl, 2) perfluoroalkyl, and 3) terminated dimethyl-silicone. For the alkyl chain, carbon atoms act as electron acceptors, while hydrogen atoms serve as electron donors. In contrast, for CF_2 of the perfluoroalkyl, the trend is reversed, with fluorine exhibiting higher electronegativity and electrons being transferred from carbon to fluorine atoms. Notably,

the comparison of the net charge distribution of CH_3 and CF_3 as terminal moieties reveals a similar charge distribution, which may suggest comparable interactions with water. Surface charge analysis demonstrates how the terminated dimethyl-silicone chain can be more negatively charged than perfluoroalkyls, which explains why the hydrogen atoms of water molecules remain at closer distances. It is noteworthy that the absolute values of partial charges in Hirshfeld analysis are not physically meaningful. Instead, they provide a qualitative measure of charge distribution and accumulation trends.[15] The average molecular lengths of the perfluoroalkyl and dimethyl-silicone chain are approximately 0.89 nm and 0.78 nm, respectively.

References

(1) Jin, Y.; Yang, S.; Sun, M.; Gao, S.; Cheng, Y.; Wu, C.; Xu, Z.; Guo, Y.; Xu, W.; Gao, X.; et al. How liquids charge the superhydrophobic surfaces. *Nat. Commun.* **2024**, *15*, 4762.
(2) Geerlings, P.; De Proft, F.; Langenaeker, W. Conceptual density functional theory. *Chem. Rev.* **2003**, *103*, 1793-1874.
(3) Hutter, J.; Iannuzzi, M.; Schiffmann, F.; VandeVondele, J. Cp2k: Atomistic simulations of condensed matter systems. *Wiley Interdiscip. Rev. Comput. Mol. Sci.* **2014**, *4*, 15-25.
(4) Perdew, J. P.; Burke, K.; Ernzerhof, M. Generalized gradient approximation made simple. *Phys. Rev. Lett.* **1996**, *77*, 3865-3868.
(5) VandeVondele, J.; Hutter, J. Gaussian basis sets for accurate calculations on molecular systems in gas and condensed phases. *J. Chem. Phys.* **2007**, *127*, 114105.
(6) Krack, M. Pseudopotentials for h to kr optimized for gradient-corrected exchange-correlation functionals. *Theor. Chem. Acc.* **2005**, *114*, 145-152.
(7) Grimme, S.; Antony, J.; Ehrlich, S.; Krieg, H. A consistent and accurate ab initio parametrization of density functional dispersion correction (dft-d) for the 94 elements h-pu. *J. Chem. Phys.* **2010**, *132*, 154104.
(8) Grimme, S.; Ehrlich, S.; Goerigk, L. Effect of the damping function in dispersion corrected density functional theory. *J. Comput. Chem.* **2011**, *32*, 1456-1465.
(9) Bussi, G.; Donadio, D.; Parrinello, M. Canonical sampling through velocity rescaling. *J. Chem. Phys.* **2007**, *126*, 014101.
(10) Martínez, L.; Andrade, R.; Birgin, E. G.; Martínez, J. M. Packmol: A package for building initial configurations for molecular dynamics simulations. *J. Comput. Chem.* **2009**, *30*, 2157-2164.
(11) Stukowski, A. Visualization and analysis of atomistic simulation data with ovito–the open visualization tool. *Model. Simul. Mat. Sci. Eng.* **2010**, *18*, 015012.
(12) Giovambattista, N.; Debenedetti, P. G.; Rossky, P. J. Effect of surface polarity on water contact angle and interfacial hydration structure. *J. Phys. Chem. B.* **2007**, *111*, 9581-9587.
(13) Chen, W.; Sanders, S. E.; Özdamar, B.; Louaas, D.; Brigiano, F. S.; Pezzotti, S.; Petersen, P. B.; Gaigeot, M.-P. On the trail of molecular hydrophilicity and hydrophobicity at aqueous interfaces. *J. Phys. Chem. Lett.* **2023**, *14*, 1301-1309.
(14) Pezzotti, S.; Serva, A.; Sebastiani, F.; Brigiano, F. S.; Galimberti, D. R.; Potier, L.; Alfarano, S.; Schwaab, G.; Havenith, M.; Gaigeot, M.-P. Molecular fingerprints of hydrophobicity at aqueous interfaces from theory and vibrational spectroscopies. *J. Phys. Chem. Lett.* **2021**, *12*, 3827-3836.
(15) Marenich, A. V.; Jerome, S. V.; Cramer, C. J.; Truhlar, D. G. Charge model 5: An extension of hirshfeld population analysis for the accurate description of molecular interactions in gaseous and condensed phases. *J. Chem. Theory Comput.* **2012**, *8*, 527-541.
